# An exploratory study on support for caregivers of people with vision impairment in the UK

**DOI:** 10.1111/opo.12989

**Published:** 2022-04-13

**Authors:** Jamie Enoch, Christine Dickinson, Judith Potts, Ahalya Subramanian

**Affiliations:** ^1^ Division of Optometry and Visual Sciences, School of Health Sciences, City University of London London UK; ^2^ Division of Pharmacy & Optometry University of Manchester and the Manchester Academic Health Sciences Centre Manchester UK; ^3^ Esme's Umbrella London UK

**Keywords:** caregivers, low vision, mixed‐methods research

## Abstract

**Purpose:**

Many of the UK's 2.5 million individuals living with vision loss receive support from relatives or friends (so‐called ‘informal caregivers’). However, there is limited understanding of how caregivers of people with visual impairment (PVI) are, or feel, supported by UK healthcare/statutory services and charities. This exploratory study was conducted to explore caregivers' experiences and their suggestions for enhancing support.

**Methods:**

Participants self‐identifying as UK‐based caregivers of PVI (*N* = 100) volunteered to undertake an online survey, distributed through charity partners. The survey was comprised of the Client Satisfaction Questionnaire‐8 (CSQ‐8, a validated, self‐report measure of satisfaction with support services), Likert‐type questions and two open‐ended, free‐text questions. Interview participants (*N* = 22) were then selected from survey respondents, and semi‐structured interviews were conducted to focus on caregivers' ideas for improving support. The Framework Method was used for inductive analysis of the free‐text question responses and interview data.

**Results:**

The mean (SD) CSQ‐8 score was 21.60 (7.2), with no significant differences by demographic, relationship or vision‐related factors, likely limited by the small subgroup sizes. Qualitative data demonstrated the heterogeneity of participating caregivers' experiences, highlighting the importance of personalised support for caregivers. Many participants advocated enhancing informational, practical, emotional and social support for caregivers, and stressed the importance of accessible services and consistent points of contact to turn to for support and advice.

**Conclusions:**

Although our sample was arguably better connected to support services than the general caregiver population, this study identified concrete suggestions to improve practical, emotional and peer support for caregivers of PVI.

**Key points**

- Previous research has documented increased levels of distress among some relatives and friends (‘informal caregivers’) of people with visual impairment and chronic eye conditions. Many caregivers of people with visual impairment in the UK receive little or no support or information, according to studies involving parents of children newly diagnosed with visual impairment and caregivers of adults with neovascular age‐related macular degeneration.
- In this exploratory study, many participating caregivers of people with visual impairment highlighted the uneven and inaccessible nature of support services, and recommended improved informational, practical, emotional and social support.
- High‐quality support for the caregiver is often contingent on people with visual impairment themselves having access to appropriate support. Parents/carers of children with visual impairment were particularly distressed by delays and bureaucracy when seeking support for their child.

## INTRODUCTION

Many of the 2.5 million individuals living with visual impairment (VI) in the UK^1^ receive regular support from family members or friends, sometimes referred to as ‘informal caregivers’. These caregivers may require support themselves, to cope with some of the more negative impacts of caregiving.^2^, ^3^, ^4^, ^5^ While the scientific literature documents the challenges experienced by caregivers of people with visual impairment (PVI), much less is known about what works to support these caregivers.^6^ A small number of studies have formally evaluated different kinds of interventions or strategies that may be most useful for caregivers of PVI. Some of these are tailored to specific groups of family members of people with sight loss, for example parents,^7^, ^8^, ^9^, ^10^ spouses or partners,^11^ or with a broader focus on caregivers more generically.^12^, ^13^ Two large‐scale randomised controlled trials that involve caregivers of people with age‐related macular degeneration (AMD)—though not necessarily all with VI—are currently ongoing.^14^, ^15^ The majority of interventions involving caregivers of PVI, such as through structured support groups or psychoeducational programmes, can help to improve knowledge and awareness of VI.^6^ Furthermore, structured support, education or training interventions for parents of children with VI demonstrate long‐term benefits in reducing parental stress.^7^, ^8^, ^9^

To some extent, interventions to support caregivers have been informed by the scientific literature documenting concerns and difficulties experienced by caregivers of PVI. Studies exploring aspects of caregiver distress conclude with recommendations for practice, including suggestions that caregivers may benefit from psychosocial interventions to boost problem‐solving skills,^2^ or referral for psychological therapy.^16^ However, there is relatively little published literature regarding the precise day‐to‐day challenges that caregivers of PVI may face, and especially little on what they would like to see change or improve.

A small number of studies have sought to document empirically the support needs of relatives and caregivers of people with VI. For example, Rahi and colleagues considered the experiences and needs of UK parents at the time when their children were diagnosed with conditions causing VI.^17^ Satisfaction with care was higher among parents whose children had mild VI or isolated ophthalmic conditions, compared with more severe VI (*p* = 0.003) or multiple impairments (*p* = 0.02). The authors also found a high level of unmet need for information about eye conditions, social services, support networks and education. Similarly, McDowell's multi‐country survey on the experiences of parents of children with cerebral visual impairment demonstrated the empowering potential of accessible, relevant information for parents about the condition and its consequences for their child.^18^ Additionally, Jackel et al. conducted a survey in the USA with parents of children with cortical or cerebral visual impairment.^19^ They showed that the experience of these parents was relatively polarised: 42% of participating parents reported receiving all necessary services, while 35% had received no support at all. Furthermore, Gohil and colleagues have explored the satisfaction of caregivers of people with AMD regarding care and support received from health services.^20^ In the latter study, most caregivers reported receiving little or no support from healthcare providers. Eighty‐two percent reported being ‘not at all’ satisfied with both the caregiver support and practical advice given. A broader issue identified across these studies is that caregivers often express a need for better information about the condition of the PVI, even when information is apparently available.^6^

Therefore, there are already a small number of studies considering how relatives or caregivers of PVI are currently receiving support. However, they largely tend to evaluate satisfaction with support, and unmet needs, quantitatively. To the best of our knowledge, no study has used a qualitative methodology to explore inductively what support (from healthcare, social or voluntary services) caregivers or relatives of PVI would find most useful. Where qualitative studies have been undertaken with caregivers of PVI, they have tended to focus on exploring their lived experiences.^21^, ^22^, ^23^ Such studies can valuably identify gaps where additional support may be needed, but they do not explore the caregivers' recommendations for further support directly.

Therefore, we decided to undertake a qualitatively driven^24^ exploratory study with a heterogeneous group of UK‐based relatives and caregivers of PVI, to consider caregivers' support needs and experiences and crucially their ideas for improving support in the UK. We structured the study around the following research questions:

1. What support have caregivers of PVI accessed, and how satisfied are they with this support?
2. Does the support accessed and level of satisfaction differ by socio‐demographic or vision‐related variables, such as the relationship between caregiver and PVI, or level of visual impairment?
3. How do caregivers of PVI themselves think that support services could be improved?

## METHODS

The study used a survey and semi‐structured interviews, to explore access to, and satisfaction with, support among a broad group of self‐identified UK‐based relatives, friends or caregivers of people with VI, and to consider their ideas for improving support services.

### Development of the survey and follow‐up interview procedure

A cross‐sectional survey was designed with four sections (shown in full in Appendix S1).

1. The first section asked caregivers to think about support they have received, and then respond to items from the Client Satisfaction Questionnaire‐8 (CSQ‐8).^25^ The CSQ‐8 is a validated questionnaire, which has been used extensively in evaluation of health and care services to measure satisfaction with services; scores range from 8 (minimum) to 32 (maximum).
2. In the second section, with kind permission from Gohil and colleagues,^20^ we reproduced a (non‐validated) questionnaire for caregivers to self‐report on the support they had received over the last 12 months, and where they tend to turn for support. The questions were either Yes/No or Likert‐type (e.g., ‘Not at all’, ‘Some’ and ‘Often’).
3. The third section involved demographic information about the caregiver, for example their age, gender, ethnicity, relationship to the person with VI and the cause and severity of the VI.
4. The fourth, optional section asked participants to share free‐text feedback, where they could elaborate on their responses to previous questions and provide additional thoughts about their experience as a caregiver. Finally, participants were asked if they might be willing to take part in a follow‐up interview.

After initial compilation of the survey and drafting of the participant information sheet, the authors consulted with an advisory group consisting of individuals with VI and caregivers to ensure information and questions were clear and appropriately phrased. The authors also obtained the advisory group's views on important open‐ended questions to ask in the follow‐up interviews.

The study received approval from the City, University of London, School of Health Sciences' Optometry Research Ethics Committee (reference ETH1920‐0009). The final version of the questionnaire was distributed online via the Qualtrics survey platform (qualtrics.com) (with the option to respond by mail or telephone) from October 2019 to March 2020, and publicised by relevant UK‐based professional bodies and charity partners in online and print media (please see Acknowledgements). The survey was open to UK‐based participants aged 18 or over who considered themselves to be a relative, friend or caregiver of a PVI. Participants read an information sheet and provided written consent before completing the survey.

The interview topic guide, used to interview a subset of survey respondents, is shown in Appendix S2. Interview participants were selected using a maximum variation sampling technique to target a mix of individuals with differing characteristics as reported in the survey.^26^ Semi‐structured interviews were conducted by telephone, after receiving reconfirmed written consent. Issues noted in the survey were explored in greater depth; therefore, while the topic guide was a useful prompt, the direction of the interview differed depending on the participants' context and experiences.

### Quantitative data analysis

The main quantitative measure was the CSQ‐8 score. CSQ‐8 scores were analysed as the main dependent variable against categorical variables (e.g., if caregiver needs had been assessed in the previous 12 months, and demographic characteristics). Statistical differences on the CSQ‐8 by group were tested using Mann–Whitney U tests (when comparing two groups) and Kruskal–Wallis tests (when comparing three or more groups). A *p*‐value of ≤0.05 was considered statistically significant, and Bonferroni's correction was applied to adjust for the multiple comparisons. Statistical tests were conducted using SPSS, version 25.0 (IBM, ibm.com).

### Qualitative data analysis

Qualitative data were collected through an open‐ended, free‐text question at the end of the CSQ‐8, about what would most help participants in their caregiving role; a free‐text box in the final section of the survey and 22 audio‐recorded interviews with selected survey participants. Detailed notes were made during interviews and on listening to the recordings, and interview extracts were transcribed verbatim.

To analyse these varied sources of qualitative data, the Framework Method,^27^ was used. Following familiarisation with the qualitative data, involving several readings and initial annotations, the first author (JE) undertook open coding inductively, generating codes to refer to and classify relevant, meaningful aspects of the data. After developing and refining these initial codes, they were grouped into higher‐order categories. The categories and constituent codes were then discussed with the senior researchers on the study (authors CD and AS) and finalised into an analytical framework. The software NVivo 12 Pro (QSR International, qsrinternational.com) was used to sort the qualitative data into this analytical framework, and subsequently produce a framework matrix, with each row representing a ‘case’ or participant, and each column a ‘code’. Finally, we interpreted the matrix as a whole, in order to generate themes. These themes aimed to encapsulate and explain meaningful patterns across the dataset, while maintaining a focus on the individual, divergent experiences of different participants.

## RESULTS

### Quantitative findings

One hundred participants responded to the survey, 98 (%) online and two by mail. Table 1 shows survey results where there were statistically significant differences in CSQ‐8 score by group. Full demographic information of participants, and their CSQ‐8 score by sub‐group, is shown in Appendix S3.

**TABLE 1:** Client Satisfaction Questionnaire‐8 (CSQ‐8) scores, overall and disaggregated against survey questions where scores differed significantly by response category

Variable	*N* (= %)	CSQ‐8 score: Scores can range from minimum 8 to maximum 32 mean (SD)	*p*‐value
All participants	100	21.6 (7.2)	N/A
From which kinds of organisations have you received support as a person supporting a visually impaired person, if any?^a^			
Healthcare services	41	23.9 (5.1)	0.02
Charities	61	24.6 (4.6)	<0.001*
Social services	22	23.8 (5.2)	0.2
None	13	9.8 (2.5)	<0.001*
Have you been given the details of a person who you can contact if you have any questions, worries or concerns about visual impairment or caring for someone with visual impairment?			
Yes	39	24.4 (6.5)	0.003*
No	59	19.7 (7.2)	
Did not respond	2	22.0 (2.8)	
When decisions are made about the care or treatment of the person you support, do you feel that your views and needs are taken into account?			
Not at all	19	17.7 (8.0)	0.01*
Hardly ever	11	17.5 (7.3)	
Sometimes	24	22.0 (6.9)	
Often	24	24.1 (5.5)	
Always	10	25.8 (5.2)	
Did not respond	12	22.4 (7.5)	
Overall, how would you rate the level of support which you have received from the health services in the last 12 months?			
Not applicable	10	25.1 (5.2)	<0.001*
Not supported at all	32	18.1 (7.9)	
Some support	23	20.0 (5.7)	
Enough support	13	24.6 (5.6)	
Very well supported	10	28.1 (3.5)	
Did not respond	12	22.4 (7.5)	
Do you feel the support you receive could be improved?			
Not at all	11	26.8 (5.9)	<0.001*
A little	14	25.4 (5.8)	
Somewhat	20	23.1 (5.2)	
Quite a lot	18	20.9 (6.0)	
Yes, a lot	25	16.1 (7.4)	
Did not respond	12	22.4 (7.5)	

^a^ For this question, participants could select more than one response, that is all those that applied. Mann–Whitney U tests were carried out to see whether CSQ scores were significantly different among those who reported receiving support from the relevant sector, versus those who did not report any support from that sector. *P‐*values were considered significant at the Bonferroni‐corrected level of 0.0125 (based on 0.05 divided by 4, as 4 tests were carried out). Thus, caregivers who reported receiving support from healthcare services tended to have higher CSQ‐8 scores than those who did not report receiving such support, although this did not reach statistical significance (as *p* = 0.02).

* *p* < 0.05 (or Bonferroni corrected for multiple comparisons).

The mean CSQ‐8 score in our sample was 21.60 (SD: 7.21; 95% confidence interval: 20.17–23.03; Cronbach's α = 0.97). Participants receiving support from charities were significantly more positive about the support they had received (as reflected by higher CSQ‐8 scores), while, as expected, those receiving no support had significantly lower CSQ‐8 scores. Participants with details of a person to contact with questions or concerns about VI, those who felt their views were taken into account in care and treatment decisions, and those who reported feeling well supported in Section 2 of the survey, showed significantly higher CSQ‐8 scores. Expectedly, participants who reported that support services could be greatly improved showed significantly lower CSQ‐8 scores.

There were no statistically significant differences in CSQ‐8 scores by socio‐demographic characteristics (Appendix S3). Participants who reported that the person with VI they supported had an additional chronic condition showed lower CSQ‐8 scores than those without, although this did not reach statistical significance (*U* = 615, *p* = 0.09, *r* = 0.19). When causes of VI were considered, those who reported the person with VI as having a rare inherited eye disease (e.g., retinitis pigmentosa) had somewhat lower CSQ‐8 scores than those without; however, this did not reach (Bonferroni‐corrected) statistical significance (*U* = 582, *p* = 0.02, *r* = 0.23).

**FIGURE 1:**
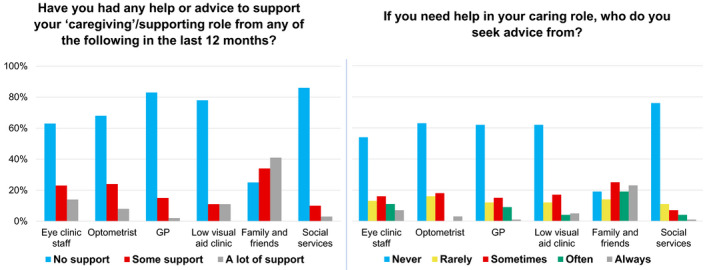
Sources of (non‐charity) support for participants. Percentages are used, since the numbers of respondents differed by question. GP, general medical practitioner

Figure 1 shows sources of support to whom participants had turned to over the last 12 months and where they are likely to go for support in future, with ‘No support’ reported most commonly, apart from in the case of support from family and friends. Study participants had also received some level of support over the last 12 months from a wide range of charities, both local and national, including Royal National Institute for Blind People (RNIB;*N* = 30), Guide Dogs (*N* = 19), Macular Society (*N* = 17), Nystagmus Network (*N* = 17), Royal Society for Blind Children (*N* = 7), Retina UK (*N* = 6), Visually Impaired Children Taking Action (VICTA;*N* = 3) and Esme's Umbrella (*N* = 2), as well as many other charities named by individual participants (e.g., Glaucoma UK, Blind Veterans, Look UK). Given the role of these charities in distributing the survey, these results cannot be considered representative or statistically meaningful (hence they are not included in Figure 1). However, knowing this context allowed us to select interview participants with varying levels of engagement with different vision charities.

### Qualitative findings

**FIGURE 2:**
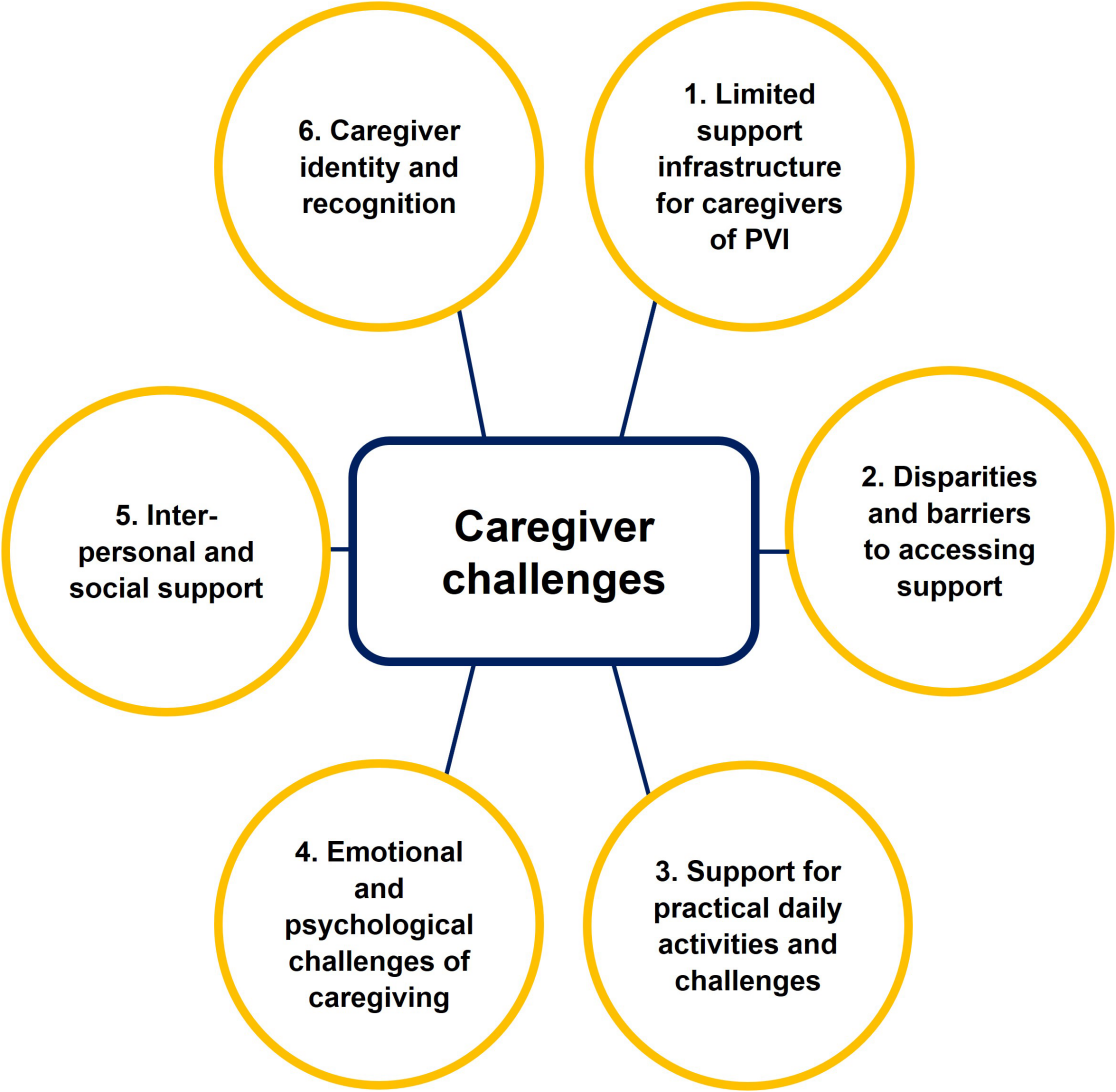
Graphic summarising themes generated from Framework Analysis of qualitative data (PVI, Person/people with visual impairment)

**FIGURE 3:**
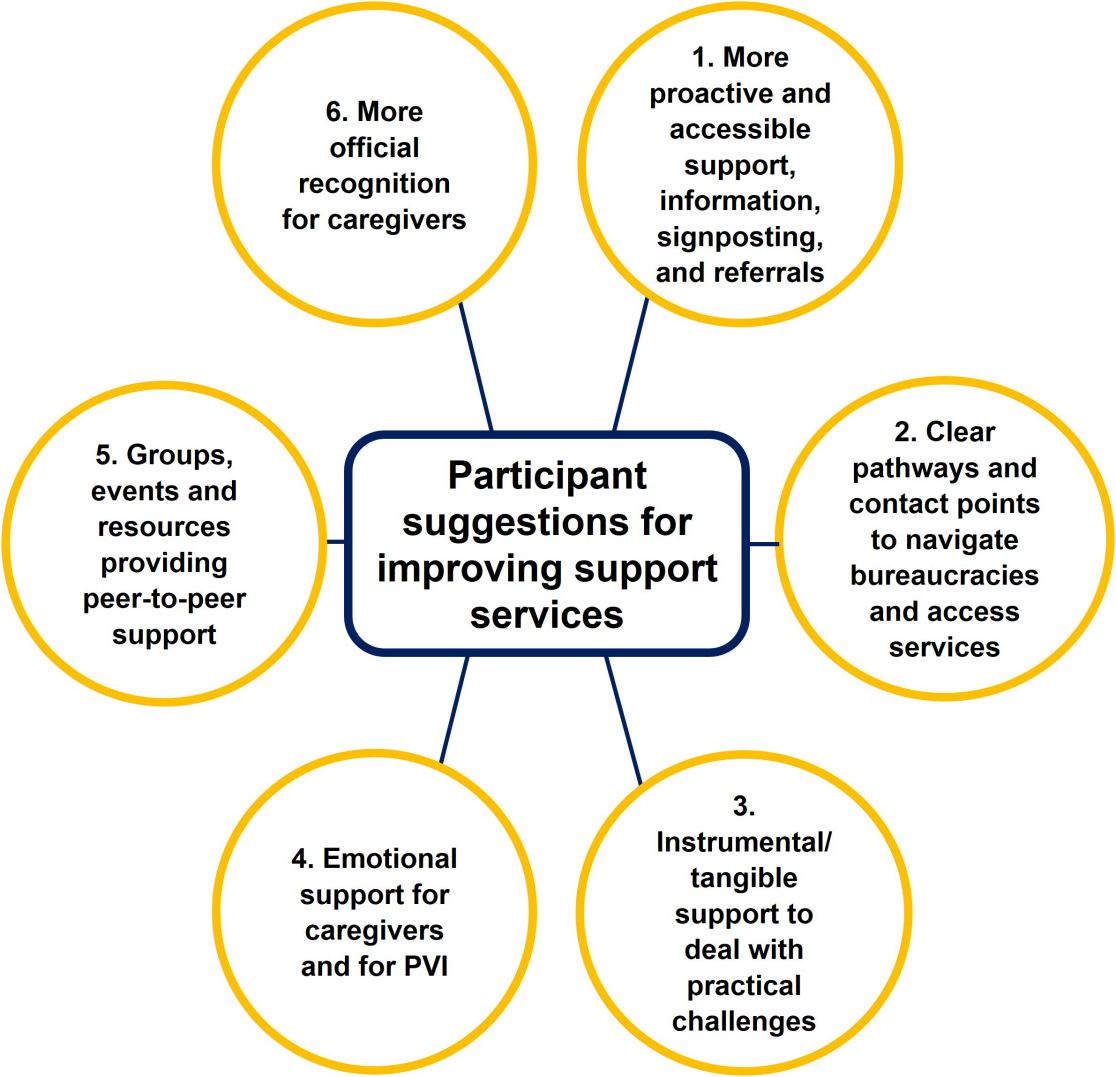
Graphic summarising participating caregivers’ suggestions for improving support services, with illustrative quotations (PVI, People with Visual Impairment)

Six themes were developed from coding and categorisation of the qualitative data, summarised in Figure 2. Table 2 shows participants’ specific suggestions for improving support services, also summarised in Figure 3. The quotation (Q) numbers refer to full quotations from interviews and survey responses, provided in the supplementary information (Appendix S4). Information in brackets after quotations refers to the person with VI, that is who they represent for the participating caregiver, and the severity of their VI; for example, if a participant is quoted as (Child, mild VI), that means the participant is the parent/caregiver of a child with mild VI.

**TABLE 2:** Summary of caregivers' suggestions for improving support services

Relevant theme	Summary of caregivers' suggestions	Illustrative quotation(s)
Theme 1: A limited support infrastructure for caregivers	Improving support for caregivers around the time when the PVI first receives a diagnosis or registration, in terms of proactive support, information and relevant signposting and referrals, before caregivers' needs become overwhelming. Trustworthy, reliable information to help caregivers understand what to expect and feel less overwhelmed, e.g., a clear, accessible standardised pack or guidance document at the point of diagnosis (ideally tailored to the relevant eye condition).	*You need more support at the beginning, it's a minefield, you've never had that in your life before* (**Spouse/partner, severe VI**). *I'd like to have walked out of that consultant's office with some information, websites, any kind of place I could go and get some information* (**Child, moderate VI**). *Information for the future of my child and how they will be able to cope* (**Child, mild VI**). *You need a ‘Steps you could expect to see’—some guidance, or a book of things of what people [with VI] will do and how to respond to it* (**Spouse/partner, moderate VI).** *A clear NHS guidance document which is given to everyone on diagnosis, pointing to the key sources of help ‐ aids, groups, websites and so on. Everything is so widely dispersed… But if somehow the NHS and charities could get together and produce a pack of some kind… you just have that information and you know what to do* (**Parent, moderate VI).** *Something written down… with links to the Macular Society or links to other charities that might be able to help and that kind of stuff. So that you've got something tangible, and you're not relying on what's been said to you* (**Spouse/partner, moderate VI**).
Theme 2: Disparities and complications accessing support	Helping caregivers to navigate bureaucracies and have better access to support services, for example through guides or flowcharts to clarify what support is available from statutory services and charities. Consistent, informed points of contact to provide advice or to signpost on for further support (e.g., an ECLO, health visitor or social worker).	*From the point where a child is diagnosed, what happens from there on?… It seems like there's no framework, no process or flowchart for this very generic, wide‐ranging disability of visual impairment. There should be a set of assessments that determine what help they need, rather than just waiting until they're eight years of age at school and they've fallen behind*… (**Child, severe VI**). *Some of the things you're not eligible for anyway. So really, you almost need some kind of flowchart to follow, to work out which kinds of things you're going to get and which you're not* (**Parent, moderate VI)**. *Someone you could sit and talk to about what it actually means in terms of what they can see, what they can't see, and what the problems might be* (**Child, mild VI**). *A regular support worker assigned to the carer and informed on the needs of the visually impaired* (**Child, severe VI**). *You need a person to call… Otherwise you got passed from pillar to post, and nobody ever knew the answer to anything* (**Sibling, moderate VI**). *Someone with the local knowledge…to point us in the right direction, to provide that single point of contact for all the services that [our child] will be needing* (**Child, severe VI**). *Someone to phone you once in a blue moon, just saying, ‘How's things now, do you need any more support?’* (**Spouse/partner, severe VI**). *In an ideal world, you need a personal social worker who turns up or is on the phone, available to you, who knows you… But it's unlikely to happen in this financially constrained health service* (**Spouse/partner, moderate VI**). *Someone to call… you might not get a result and the outcome you need, but you might get a link to it, the start of the process* (**Spouse/partner, severe VI**).
Theme 3: Support for practical day‐to‐day challenges	Instrumental support and tangible assistance to deal with practical day‐to‐day challenges, including: Fairer benefits and financial support for caregivers. Advocates to provide support with applying for benefits. Support with mobility (e.g., guiding) and transportation (e.g., befriending or buddy systems to share transport). Training in skills and knowledge potentially required in the caregiving role. Options for breaks, respite or back‐up care.	*[It would help to have] Proper financial support for assistive technology* (**Parent, severe VI**). *This chap came about 2 or 3 times and spent a total of about six hours with her, and he went through the PIP form with us… and that's probably the most help we've had* **(Child, severe VI)**. *[It would help to have] training on guiding a VI person* (**Spouse/partner, severe VI**). *He really needs to use the white cane now. And [it would help] if there were people to ask about how they got around that with their partners, or how people dealt with that situation* (**Spouse/partner, severe VI**). *It could be a volunteer or buddy who would become a friend over the years… if it got to the stage where we couldn't drive or something, just the fact that you could ring up… this person rather than a taxi company*… (**Child, severe VI**). *When Macular is diagnosed, the carer needs ‘training’ in their future role… and a method of identifying what you have and haven't got in terms of skills and knowledge gaps* (**Spouse/partner, severe VI**). *It's almost like you've got to run a course for these carers as well, so they understand that this is what you're gonna be faced with. And you could get people like me to speak about our experiences* (**Parent, severe VI**). *The ability to walk away, to have sanity breaks—it's the respite… It's not overnights and things like that, it's just an hour* (**Spouse/partner, moderate VI**). *When I was in hospital, it became apparent the absolute lack of support for my wife, should I not be about. Fortunately friends and neighbours rallied in… but from an official support perspective, there's literally nothing there* (**Spouse/partner, severe VI**).
Theme 4: Emotional, relational and psychological challenges of caregiving	Emotional support for caregivers, including regular ‘check‐ins’ to mitigate against distress and loneliness. Advice for caregivers on supporting the mental and emotional health of the PVI, especially if dealing with progressive, worsening vision loss.	*Certainly [there's been] no emotional support, support to check that I was coping as a human—no, I certainly didn't get any of that… Even if they just phoned. It doesn't have to be a home visit, just somebody to check in, just once a month, with the carer, to check I was surviving* (**Child, moderate VI**). *One thing is how to help [my mother] to cope with the emotional distress of having lost more sight and having worse vision, because she's extremely stressed by having lost more sight, you know, quite suddenly* **(Parent, severe VI).**
Theme 5: Interpersonal and social support	Groups, events and resources to help provide peer‐to‐peer advice and support. Many participants were keen to meet other caregivers or relatives of PVI in similar situations, both to exchange tips and also to talk in a forum where others would have a good understanding of their experience.	*A safe space for people to talk, maybe in local authorities… once a year you had a reunion* **(Spouse/partner, severe VI)**. *[I would like] someone to talk to who was in the same position as myself, but was also positive and not negative!* (**Child, severe VI**). *Receiving information on a regular basis, maybe a sort of e‐newsletter or something, directed at people who perform this role*… **(Parent, moderate VI)**. *It would be lovely to know there was some sort person I could contact or group who I could join to just chat about our personal experience and if there were ways I could better support my husband* (**Spouse/partner, severe VI**).
Theme 6: Caregiver identity and recognition	More official recognition of the caregiver's role, particularly to help when dealing with healthcare and social services. Consideration of the financial implications of caregiving.	*You're not a nominated person, the caregiver does not exist in the system—that's the main problem, isn't it… [You need] some sort of link, to get round the bureaucracy of privacy… because there is no such thing as a caregiver in the real world* (**Spouse/partner, moderate VI**). *It might be very helpful for services to know that there is a nominated person and who that might be. Obviously they would have to give their consent, but for that to be acknowledged would be quite helpful* (**Parent, moderate VI**). *[We need] recognition that caring by a family member (although given with love) has significant financial impact for the carer both in the ability to earn now and to prepare for old age with savings and pensions* (**Parent, severe VI**).

#### Theme 1: A limited support infrastructure for caregivers

Many participants had never been offered support themselves (Appendix S4, Q1–6), and several had not received ‘*any specific help as a carer*’ (Parent, moderate VI) that they could evaluate in the survey or interview (Q7–11). While participants emphasised a crucial distinction between support for themselves as a caregiver and support for the PVI, the two were often considered closely connected (Q12–14). More proactive support and referrals from ophthalmology clinics at the time of the PVI's diagnosis could help before issues became overwhelming (Q15–19), with many caregivers given the news of diagnosis without any follow‐up support. In the absence of pre‐emptive support and information from health professionals, many participating caregivers felt that the responsibility fell to them to proactively seek appropriate information and support (Q20–27), for example ‘*All too often it is the parent that has to guess the problem and then seek out the cause*’ (Child, moderate VI). Indeed, those who had received support sometimes felt ‘*very lucky*’ (Child, moderate VI) or attributed support to a helpful encounter with a specific individual, either within or outside statutory services (Q28–32).

Participants also discussed barriers to seeking‐help, because of ‘*pride*’ or feeling a pressure to ‘*manage*’ (Q33–35). A small minority of participating caregivers stated that they ‘*managed perfectly well without outside help*’ (Spouse/partner, Severe VI) (Q36–39). However, several participants reflected that their support needs could increase if their or the PVI's circumstances changed, for example due to chronic health conditions or disabilities (Q40–42).

Information was seen as a vital form of support, and the support which caregivers most commonly reported receiving; however, caregivers differentiated receiving information about managing eye conditions and living with VI from more fundamental *‘support for me as a caregiver’* (Child, moderate VI) (Q43–45). Participants also highlighted the importance of accessible, easily intelligible post‐diagnostic information, tailored, for example, to caregivers without English as a first language, and appropriate in the context of receiving what can often be a shocking, unsettling diagnosis (Q46–49). A small number of participants also raised concerns regarding the accessibility and trustworthiness of information available online (Q50–51).

In the light of these issues and experiences, participants recommended trustworthy, reliable information to help caregivers understand what to expect and to feel less overwhelmed (Table 2). Several participants suggested receiving a standardised pack or guidance document at the point of diagnosis, ‘*pointing to the key sources of help—aids, groups, websites and so on*’ (Parent, moderate VI). This guidance could be tailored to the eye condition in question, and sufficiently clear and simple to be accessible and avoid overwhelming the caregiver.

#### Theme 2: Disparities and complications accessing support

Disparities and complications accessing support were encapsulated by descriptions of the support system as ‘*a hidden maze*’ (Child, severe VI), ‘*disjointed*’ (Spouse/partner, severe VI) and ‘*labyrinthine*’ (Child, mild VI). Reaching the right service could be difficult for caregivers, often feeling that they were endlessly being referred to another agency, or confused about which services and organisations were appropriate for their situation (Q52–54). As one participant stated, ‘*Everyone seems to refer me on to someone else*’ (Parent, severe VI). In particular, parents of children with VI described being overwhelmed navigating bureaucratic systems (Q55–59), and unsure which statutory supports their child is legally entitled to. Both caregivers of children and adults with VI hoped that registration as visually impaired might unlock more support, though continued to feel the onus was on them to prompt services and push for support (Q60–61). Several participants had experienced moving between local authorities, finding substantial improvement or deterioration in support quality between areas, and limited communication across councils (Q62–68). Participants voiced frustrations at funding gaps, referring to the ‘*postcode lottery*’ (Spouse/partner, severe VI) and the short‐termist lack of support for PVI and caregivers (Q69–70), given that ‘*better support early on saves money later*’ (Child, mild VI). Often participants were more positive about support received from charities than statutory services, but were concerned about excessive pressure being placed on the voluntary sector (Q71–73), and the fact that charities may not cater so well to caregivers in rural areas or working full‐time (Q74–75).

A key recommendation to help navigate an overstretched, challenging system was a single, consistent, informed point‐of‐contact (Q76–84), who might be able to provide advice or signpost directly to the right service (Table 2): ‘*Just someone who can be a point of contact… to give that bit of support when you need it really*’ (Spouse/partner, severe VI). Some participants also suggested a *‘guide’* (Child, mild VI) or ‘*flowchart*’ (Child, severe VI) to clarify the statutory support they are legally entitled to, as well as the support available from charities.

#### Theme 3: Support for practical day‐to‐day challenges

In terms of dealing with practical day‐to‐day challenges, several caregivers noted the significant financial stress they experience (Q85–88), coupled with the costs of accessing support services (e.g., transport) (Q89–90) and the high cost of low vision aids for PVI (Q91–94). Participants also discussed complications applying for benefits such as Carer's Allowance, or Personal Independence Payment for the PVI (Q95–103). Some caregivers were struggling to balance caregiving with employment, causing intense stress, or had stopped working because of caregiving responsibilities (Q104–108).

Alongside the need for financial support, participants also discussed the need for more practical support, to respond to challenges with transportation (Q109–112), mobility (Q113–116) and a diverse range of daily activities in the home (Q117–125). Respite for caregivers—not necessarily for extended periods of time, but just ‘*having a break*’ (Spouse/partner, severe VI)—was an unmet support need (Q126–129), as well as back‐up support should anything happen to the primary caregiver.

Participating parents discussed how schools could better support the caregiver and child together (Q130–131), and the need for ‘*more support within school*’ (Child, mild VI), or at pre‐school age. Qualified teachers of the visually impaired (QTVI) provided helpful, ‘*brilliant*’ (Child, moderate VI) support to children with VI and (indirectly) to their families, though some parents had found QTVI input to be more generic and limited (Q132–135).

Findings within this theme suggest caregivers could benefit from instrumental support and tangible assistance to deal with practical day‐to‐day challenges that come in a wide range of forms. Caregivers' suggestions for improving practical support included (Table 2): one‐to‐one help with filling out application forms for financial support; financial assistance for assistive technology; a ‘*buddy system*’ (Child, severe VI) to provide local support with transportation; training courses for caregivers to learn skills to support PVI with mobility and daily activities; and opportunities for respite breaks so that caregivers can have time to themselves.

#### Theme 4: Emotional, relational and psychological challenges of caregiving

Many participants focussed on the emotional, relational and psychological challenges of caregiving, and emphasised the close, sometimes inseparable, interconnection between the PVI's well‐being and their own (Q136–139). Much of the distress experienced by caregivers was intensified by the lack of practical support for the PVI and by frustrating efforts to navigate bureaucratic systems in order to obtain support; where support for the PVI was more securely established, this greatly helped caregivers by extension (Q140–142).

A particular relational challenge was helping the PVI where required, while supporting them to remain independent (Q143–150). An additional challenge for caregivers was supporting the PVI with their mental health, especially in the case of worsening sight loss (Q151–154). While the PVI and caregiver may have closely intertwined experiences, some participants also focused on the need to consider the well‐being of the caregiver as firmly distinct from that of the PVI, and the need for support to help caregivers cope with the shock, sadness, guilt, loneliness and fatigue they may feel (Q155–158). Some caregivers, especially parents, had considered formal mental health support (e.g., counselling or psychotherapy), but expressed the difficulty of reflecting on their own feelings when so focussed on the immediate practicalities of looking after their child (Q159–162). However, other participants did suggest a need for more emotional support, even if this were just a regular check‐in from a healthcare professional (Q163–164).

Some participants highlighted a lack of understanding from others, or fear of burdening family and friends with their problems, of being *‘a nuisance for people’* (Child, severe VI). In this context, group support, for example from other caregivers of PVI or faith‐based organisations, could be helpful and reassuring (Q165–168).

Findings within this theme attest to the value of offering emotional support to caregivers, for example in terms of advice on how to cope with distress and loneliness, or regular contact from a trusted professional to ‘check in’. Several participants also expressed a wish for more help to support the mental health and wellbeing of the PVI, especially in the case of progressive vision loss (Table 2).

#### Theme 5: Interpersonal and social support

In terms of interpersonal and social support, a large number of participants emphasised the importance of meeting caregivers in similar situations (Q169–178), to exchange practical advice as well as emotional support to help caregivers realise they are ‘*not alone*’ (Spouse/partner, severe VI). Some highlighted the value of trying out several groups, in order to find the most appropriate, supportive environment. However, the rarity of certain conditions associated with VI could complicate the search for other caregivers in comparable situations (Q179–180). Other participants stressed the challenge of initially linking caregivers to groups, suggesting a system whereby a ‘*carer that's experienced*’ (Spouse/partner, severe VI) could introduce newer caregivers to relevant groups and networks (Q181–182).

Online communities, such as forums or social media groups, were found by certain participants to be invaluable platforms for sharing tips, as well as being safe spaces to be open about the challenges associated with caregiving (Q183–187). At the same time, some participants also recommended greater provision for in‐person meetings of caregivers (Q188–190), such as ‘*a safe space for people to talk, maybe in local authorities*’ (Spouse/partner, severe VI).

There was also a perceived need to improve societal awareness of VI, and tackle problematic assumptions, prejudice and lack of understanding from others about VI, especially as it is often ‘*hidden and hideable*’ (Spouse/partner, severe VI) (Q191–196).

#### Theme 6: Caregiver identity and recognition

In terms of caregiver identity and recognition, one issue encountered in the survey and interviews was the terminology of ‘caregiving’, which many participants found problematic, preferring terms such as ‘*supporter*’ or ‘*facilitator*’, or even favouring ‘*carer*’ over ‘caregiver’ (Q197–201). Many participants saw the support they provide for PVI as a natural part of being a parent, spouse or child, or as just one aspect of the mutual, reciprocal support and inter‐dependence in their relationship (Q202–207). Others felt uncomfortable or ambivalent about the term ‘caregiver’, but accepted it due to the lack of a suitable alternative or the pragmatic need to find *‘some kind of label if you're trying to talk about these people collectively*’ (Parent, moderate VI) (Q208–211). Others were accepting of the term (Q212–214), especially when dealing with statutory services and authorities. Some participants also discussed the need for caregivers to reframe their caring activity as a service for which they should receive appropriate recognition and financial support, without feeling guilty for doing so (Q215–218). Participants suggested that recognition as a caregiver or a ‘*nominated person*’ could be particularly helpful when dealing with healthcare and social services (Q219–221) (Table 2).

## DISCUSSION

This exploratory study aims to provide a snapshot of how a wide range of caregivers of PVI in the UK are currently supported by charity, healthcare and social services, and how support could be improved. Suggestions for improving support are summarised in Table 2 and Figure 3.

The quantitative results showed a lower level of satisfaction with support services than the caregivers in a study of spousal caregivers of people with AMD.^20^ In the present study, the mean CSQ‐8 score (min:8, max:32) was 21.60 (SD:7.2), compared to 28.4 (SD:4.1) in that study.^20^ We found no statistically significant differences in CSQ‐8 by socio‐demographic or vision‐related factors (Appendix S3), likely limited by the small subgroup sizes. CSQ‐8 scores were (expectedly) higher among participants who felt very well supported over the last year, had details of a person to contact with queries or concerns, felt involved in clinical decision‐making and did not think support services required improvement (Table 1).

The heterogeneity of our sample, including caregivers of PVI affected by many different kinds and degrees of sight loss, creates a challenge in generalising about caregivers' experiences and the further support required. As one participant said, ‘*you can't make a group where there isn't a group*’, expressing the difficulty of meeting caregivers of PVI in comparable situations, especially when the PVI is affected by a rare eye condition. Furthermore, in this study many participants voiced reservations about use of the term ‘caregiver’ (Theme 6), which raises questions about how far it is possible to amalgamate the participants' diverse experiences and support needs. There are parallels here with broader research involving caregivers, showing that carers may resist identification with overly formal, bureaucratised terms like ‘carer’ that do not correspond with the nuanced realities of their relationships.^28^, ^29^

Despite considerable heterogeneity, analysis of qualitative data showed recurring patterns in caregivers' experiences (Figure 2) and what they might wish to change, in terms of more personalised, accessible and timely informational, practical, emotional and peer support (Table 2, Figure 3). It is perhaps unsurprising but striking that our findings align closely with recommendations for improving support for PVI themselves, integrating emotional, informational, practical and peer support,^30^, ^31^ and for developmental advice and emotional support for parents of children with VI.^32^ It was also apparent that support for caregivers cannot be neatly demarcated from support for the PVI, and that one fundamental route to supporting caregivers is through holistic and joined‐up structures of support for the PVI, that are also inclusive of the caregiver. Frequently, adequate support for the PVI was a prerequisite for caregivers to feel supported themselves. Consequently, participants often opted to discuss support for the PVI when asked about what support would be most helpful for them as caregivers. Furthermore, many participants were unaware that support services existed for caregivers, having witnessed the limited support available for the PVI, or felt that they were unlikely to benefit from what was available.

Many articles documenting burden and stress among caregivers of PVI suggest the need for further mental health support, and this similarly emerged in some participants' accounts. Nonetheless, while counselling could be beneficial for certain caregivers, for others psychological support was not necessarily a solution when they were so focused on attending to immediate, practical concerns. This finding is in line with Cutrona's theory of social support, which suggests that when stressful situations can be addressed with problem‐solving, instrumental support (such as information and tangible resources) may be more beneficial than emotional support.^33^, ^34^ Emotional support may instead be more helpful when events or circumstances cannot be changed. At the same time, the quality as well as the type of support intervention—the ‘process’ rather than ‘content’ of support—is important, since instrumental support has been shown to be most beneficial for well‐being when provided with empathy and understanding.^35^ This could explain why peer groups—where caregivers could exchange advice and share their experiences with other ‘*people in the same boat*’ (Spouse/partner, moderate VI) in a supportive, empathetic environment—were so strongly endorsed by the participants. Peer groups can effectively combine instrumental/practical and emotional support,^36^, ^37^, ^38^ although naturally there will be differences in individuals' attitudes towards and engagement with such groups,^39^ and quantitative findings on the benefits of peer support are more equivocal.^40^, ^41^ Many participants highlighted that some groups will have more beneficial aspects than others, for example specifically tailored to parents/carers of children with one kind of eye condition, or simply with a different structure or atmosphere. Therefore, future research could valuably explore the potential benefits and most effective modes of peer support and befriending for caregivers of PVI.

Research suggests there are common aspects of the caregiving experience across chronic conditions, and points to the importance of more circumstantial or psychosocial factors, such as structural advantages (e.g., financial resources), relationship quality and caregiving coping strategies.^42^, ^43^, ^44^, ^45^ Indeed, our findings have parallels with results from other studies involving UK‐based caregivers, relatives or parents of people living with chronic illnesses or disabilities. Such studies often point to the challenges UK‐based caregivers face navigating bureaucratic systems to access appropriate support, for example dealing with complex forms to apply for social security benefits^46^ within a disjointed,^47^ ‘maze’‐like system of health, social care and voluntary agencies.^48^ Therefore, many of the support needs of caregivers of PVI are not unique to vision, and so may seem to go beyond the remit of eye care professionals. However, awareness of the issues that caregivers face may help with referrals and signposting to more generic support services outside eye care. Furthermore, given participants' emphasis on consistent, central points of contact who can provide advice, support and onward referrals or signposting, Eye Clinic Liaison Officer (ECLO) service provision could be scaled up to ensure more comprehensive coverage for caregivers and families.^49^, ^50^ At the same time, in a financially constrained low vision and social care system and overstretched voluntary sector, it should be noted that responding to our participants' suggestions for improving support (Table 2, Figure 3) did not invariably imply a need for new services. Many suggestions related to better organising and consolidating of what is already available; for example, ensuring that at the point of diagnosis, information about the relevant condition and onward support services are proactively provided by ophthalmologists.^10^, ^18^ Furthermore, it is clear that many caregivers felt their distress could be alleviated by improving societal awareness of VI, and challenging widespread prejudice and discrimination against PVI. This points to the value of advocacy and campaigns that seek to dismantle social, environmental and economic barriers faced by PVI.

Our survey and interviews concluded before COVID‐19 restrictions came into force in the UK, and it will be important to consider how the pandemic may have changed and increased demands on caregivers of PVI. A Fight for Sight report released in September 2020, taking the pandemic's impacts into account, shows significant thematic overlap with our findings, for example highlighting how the government ‘must acknowledge the role of informal carers [and] provide them with the necessary financial and respite support’^1^ (for which carer organisations have long been advocating).^51^ Indeed, the pandemic is thought to have had negative psychosocial impacts on people with VI,^52^ and given our findings about the interconnected wellbeing of the caregiver and PVI, there may have been secondary impacts on caregivers. With many ophthalmology and low vision services being reconfigured to run remotely,^53^, ^54^ it will be important to explore how caregivers as well as PVI themselves can best be supported remotely. Notably, in our (pre‐pandemic) study, participants strongly endorsed occasional telephone ‘check‐ins’ from professionals, which would be fully compatible with social distancing regulations.

This exploratory study has several limitations. Firstly, our recruitment strategy through vision charities inevitably introduced selection bias, since our volunteer participants were arguably better connected to charity support services than most caregivers of PVI. Nonetheless, caregivers' overall level of satisfaction with support (as measured by the CSQ‐8) ranged widely, and qualitative responses attested to gaps in support and the need for significant improvements. Equally, there is the possibility of selection bias in the opposite direction, with caregivers most significantly impacted and invested in improving support services perhaps feeling more motivated to participate. Ultimately, without data on non‐responders, this remains uncertain. A second limitation of our study was sampling bias resulting in underrepresentation of Black, Asian and minority ethnic participants; 97.5% of the participants who reported their ethnicity in the present study were white. This speaks to the importance of further research involving Black, Asian and minority ethnic caregivers, given that caregiving literature in other fields (such as dementia) has documented specific barriers which may impede access to support for caregivers from minority ethnic communities.^55^, ^56^ A third limitation relates to the computer literacy of our participants, with all but two participants completing the survey online rather than by post or telephone. Participants were on average highly educated, with 46% reporting having studied to degree level (compared to an average of 42% for 21–64 year olds in UK).^57^ Thus, there are limits to how far the experiences discussed here can generalise to all caregivers of PVI in the UK, and to other geographical contexts. This underscores the importance of further research to understand the experiences of support services and unmet needs for more specific sub‐groups of caregivers of PVI.

Despite these limitations, a key strength of this study is empirically documenting caregivers' ideas for how support could be improved practically. The findings clearly confirm that providing informal care or support for relatives, with VI or any condition, is ‘a subjective experience… not a one‐size‐fits‐all phenomenon’,^58^ a dynamic trajectory which, in the words of one participant ‘*twists and turns in its journey*’. In summary, the present study suggests that while caregivers' support needs are closely linked to those of the PVI, caregivers of PVI often experience their own challenges navigating a disjointed system and many could benefit from more holistic, personalised support in their own right. This includes more accessible information, clearer pathways and contact points to navigate the support system, and a range of tangible, emotional and peer support, as well as more official recognition of caregivers' roles (Table 2/Figure 3). Further research could helpfully trial interventions to respond to these unmet support needs, while also integrating outcome measures for caregivers into research involving PVI.

## CONFLICT OF INTEREST

Jamie Enoch, Christine Dickinson, Judith Potts and Ahalya Subramanian have no interests to declare.

## AUTHOR CONTRIBUTIONS

**Jamie Enoch:** Data curation (lead); formal analysis (lead); investigation (equal); methodology (equal); project administration (supporting); writing – original draft (lead). **Chris Dickinson:** Conceptualization (lead); formal analysis (supporting); funding acquisition (lead); project administration (lead); supervision (equal); writing – original draft (supporting); writing – review and editing (lead). **Judith Potts:** Conceptualization (equal); methodology (equal); validation (equal); writing – review and editing (equal). **Ahalya Subramanian:** Conceptualization (lead); formal analysis (supporting); funding acquisition (lead); investigation (equal); methodology (equal); project administration (lead); supervision (lead); writing – original draft (supporting); writing – review and editing (lead).

## Supporting information


Appendix S1

Appendix S2

Appendix S3

Appendix S4
