# Supplementary material for: An exploratory study on support for caregivers of people with vision impairment in the UK
Source: Ophthalmic Physiol Opt. 2022 Apr 13;42(4):858–71. doi: 10.1111/opo.12989 (PMC9320821; doi:10.1111/opo.12989)
Supplement: Supplementary file 1 — Appendix S1 [file OPO-42-858-s002.docx]

# Appendix 1: Copy of survey instrument

**Part 1: Client Satisfaction Questionnaire-8 (CSQ-8)**

The CSQ-8 questions cannot be reproduced here due to copyright restrictions. Please see Attkisson and Zwick (1982)^21^ for further information about the CSQ-8.

**Part 2: Caregiver support questionnaire (With kind permission from Gohil et al, 2015)**

| **1.** Have you had any help or advice to support your ‘caregiving’/supporting role from any of the following **in the last 12 months**? | | | | | | | |
| --- | --- | --- | --- | --- | --- | --- | --- |
| a. **Eye Clinic Staff (e.g. doctors, Eye Clinic Liaison officers)** | 1  No support | | 2  Some support | | | 3  A lot of support | |
| b. **Optometrist** | 1  No support | | 2  Some support | | | 3  A lot of support | |
| c. **GP** | 1  No support | | 2  Some support | | | 3  A lot of support | |
| d. **Low visual aid clinic** | 1  No support | | 2  Some support | | | 3  A lot of support | |
| e. **Family and friends** | 1  No support | | 2  Some support | | | 3  A lot of support | |
| f. **Macular Disease Society** | 1  No support | | 2  Some support | | | 3  A lot of support | |
| g. **RNIB** | 1  No support | | 2  Some support | | | 3  A lot of support | |
| **h. Retina UK** | 1  No support | | 2  Some support | | | 3  A lot of support | |
| **i. International Glaucoma Association (IGA)** | 1  No support | | 2  Some support | | | 3  A lot of support | |
| **j. Royal Society for Blind Children (RSBC)** | 1  No support | | 2  Some support | | | 3  A lot of support | |
| **k. Esme’s Umbrella (for Charles Bonnet Syndrome)** | 1  No support | | 2  Some support | | | 3  A lot of support | |
| **l. Guide Dogs for the Blind Association** | 1  No support | | 2  Some support | | | 3  A lot of support | |
| **m. Other low vision or sight loss charity** (Please specify: _____) | 1  No support | | 2  Some support | | | 3  A lot of support | |
| **n.**  **Social services** | 1  No support | | 2  Some support | | | 3  A lot of support | |
| **o.** **Other** (Please specify):  ____________________ | 1  No support | | 2  Some support | | | 3  A lot of support | |
| **2.** Have you been given the details of a person who you can contact if you have any questions, worries or concerns about visual impairment or caring for someone with visual impairment?  YES NO  **3.** Have your needs as a caregiver been assessed by any health professional **in the last 12 months**?  If ‘Yes’, who undertook the assessment?  Yes ________________ No | | | | | | | |
| **4.** When decisions are made about the care or treatment of the person you support, do you feel that your views and needs are taken into account? | 1  Not at all | 2  Hardly ever | | 3  Sometimes | 4  Often | | 5  Always |
| **5.** Overall, how would you rate the level of support which you have received from the health services **in the last 12 months**? | 1  Not supported at all | 2  Some support | | 3  Enough support | 4  Very well supported | | 5  Not applicable |
| **6.** Do you feel the support you receive could be improved? | 1  Not at all | 2  A little | | 3  Somewhat | 4  Quite a lot | | 5  Yes, a lot |
| **7.** If you need help in your caring role who do you seek advice from? | | | | | | | |
| a. **Doctors in Eye Clinic** | 1  Never | 2  Rarely | | 3  Sometimes | 4  Often | | 5  Always |
| b. **Optometrist** | 1  Never | 2  Rarely | | 3  Sometimes | 4  Often | | 5  Always |
| c. **GP** | 1  Never | 2  Rarely | | 3  Sometimes | 4  Often | | 5  Always |
| d. **Low visual aid clinic** | 1  Never | 2  Rarely | | 3  Sometimes | 4  Often | | 5  Always |
| e. **Family and friends** | 1  Never | 2  Rarely | | 3  Sometimes | 4  Often | | 5  Always |
| f. **Macular Disease Society** | 1  Never | 2  Rarely | | 3  Sometimes | 4  Often | | 5  Always |
| g. **RNIB** | 1  Never | 2  Rarely | | 3  Sometimes | 4  Often | | 5  Always |
| h. **Retina UK** | 1  Never | 2  Rarely | | 3  Sometimes | 4  Often | | 5  Always |
| i. **International Glaucoma Association (IGA)** | 1  Never | 2  Rarely | | 3  Sometimes | 4  Often | | 5  Always |
| j. **Royal Society for Blind Children (RSBC)** | 1  Never | 2  Rarely | | 3  Sometimes | 4  Often | | 5  Always |
| k. **Esme’s Umbrella (for Charles Bonnet Syndrome)** | 1  Never | 2  Rarely | | 3  Sometimes | 4  Often | | 5  Always |
| l. **Guide Dogs for the Blind Association** | 1  Never | 2  Rarely | | 3  Sometimes | 4  Often | | 5  Always |
| m. **Other low vision or sight loss charity** (Please specify: _____) | 1  Never | 2  Rarely | | 3  Sometimes | 4  Often | | 5  Always |
| n.  **Social services** | 1  Never | 2  Rarely | | 3  Sometimes | 4  Often | | 5  Always |
| o. **Other** (Please specify):  ____________________ | 1  Never | 2  Rarely | | 3  Sometimes | 4  Often | | 5  Always |

**8**. How do you have contact with health professionals? (Please select all that apply):

Face to face Telephone E-mail In a group

**Part 3: Demographic questions**

- Are you…?
- 18 – 24
- 25 – 34
- 35 – 44
- 45 – 54
- 55 – 64
- 65 – 74
- 75 – 84
- Over 85
- I would rather not say
- What is your gender?
- Female
- Male
- Other (please specify): _______________
- I would rather not say
- How would you describe your ethnic background?
- Asian or Asian British - Bangladeshi
- Asian or Asian British - Indian
- Asian or Asian British - Pakistani
- Black or Black British - African
- Black or Black British – Caribbean
- Black or Black British - Other Black
- Chinese
- Mixed White and Asian
- Mixed White and Black - African
- Mixed White and Black - Caribbean
- Mixed Other
- Other White
- White British
- White Irish
- Other Ethnic Group
- I would rather not say
- What is your marital status?
- Single
- Married
- Living with partner
- Divorced
- Widowed
- I would rather not say
- From the following options, please indicate the highest level of education you have achieved so far:
- No formal qualifications
- GCSE or equivalent
- A Level or equivalent
- Undergraduate degree (Bachelors degree or equivalent)
- Postgraduate degree (Masters, PhD or equivalent)
- Other (please specify): _____________________
- I would rather not say
- What best describes your religion or belief?
- Buddhist
- Christian
- Hindu
- Jain
- Jewish
- Muslim
- Sikh
- No religion
- Other (please specify)
- I would rather not say
- Do you consider English to be your first language?
- Yes
- No
- I would rather not say
- Do you currently suffer from any chronic (long-term) health condition(s)?
- Yes
- No
- I would rather not say

If yes, please specify the condition: _________________

- Please provide the first part of your UK postcode (e.g. EC1, M13): ____________
- Is the visually impaired person you support/care for your…?
- Spouse/partner
- Parent
- Child
- Sibling
- Friend/neighbour
- Other (please specify) _____________
- Does the person you support/care for have…?
- Mild visual impairment
- Moderate visual impairment
- Severe visual impairment
- I do not know
- Is the person you support/care for…?
- Legally certified as severely sight impaired (“blind”)
- Legally certified as sight impaired (“partially sighted”)
- Not certified
- I do not know
- Does the person you support/care for have any of the following conditions that are linked to visual impairment (please tick all that apply)?
- Age-related macular degeneration (AMD)
- Cataracts
- Cerebral visual impairment
- Charles Bonnet Syndrome
- Childhood ocular blindness
- Diabetic Retinopathy
- Eye injuries or infections
- Glaucoma
- Neurological diseases (e.g. visual impairment after stroke or trauma)
- Rare inherited eye diseases (e.g. Retinitis pigmentosa, Leber congenital amaurosis, Stargardt disease)
- Other - please specify: __________
- Does the person you support/care for have another chronic (long-term) condition as well as their visual impairment?
- Yes
- No
- I would rather not say

If yes, please specify the condition: _________________

**Part 4: Additional feedback**

**If you have comments relating to the questions above, or any thoughts you’d like to share about your experience as a ‘caregiver’ or person supporting someone with a visual impairment, please write these in the box below:**

**Would you be willing to take part in a telephone interview to discuss your experiences, and your thoughts about improving support for ‘caregivers’ or persons supporting visually impaired people? (If YES, we will need your contact details.)**

YES / NO

**Would you like to be entered into our prize draw? (If YES, we will need your contact details.)**

YES / NO

**Would you like to be informed of the study results? (If YES, we will need your contact details.)**

YES / NO

**If you have answered YES to any of the questions above**, please provide the following details:

Name: _______________________________

Telephone number: _______________________________

Email address: _______________________________

*We will only use these details to contact you for the purpose you have indicated – your details will not be used for anything else, and we will not pass them on.*
