# Supplementary material for: An exploratory study on support for caregivers of people with vision impairment in the UK
Source: Ophthalmic Physiol Opt. 2022 Apr 13;42(4):858–71. doi: 10.1111/opo.12989 (PMC9320821; doi:10.1111/opo.12989)
Supplement: Supplementary file 2 — Appendix S2 [file OPO-42-858-s003.docx]

# Appendix 2: Semi-structured interview topic guide

- How did you find filling out the survey?
- Were there questions on the survey that surprised or stood out for you?
- Please tell me a bit more about your experience of providing care/support for your family member.
  - Has the amount of help or the type of help your family member and you have required changed over time?
  - When your family member was diagnosed with VI, did you expect the experience of caring for them to be like this?
  - Has what you expected actually happened (or are you caring for them more or less than you thought?)
- Were you offered any support at the time of diagnosis?
  - Through the health service? Through charities?
  - Would you have known at the time of diagnosis what sort of support you might need?
  - *Follow-up questions*: What kind of support? By whom? For how long? Did you request support, or was it offered automatically? Was it helpful?
- If you were never offered support at diagnosis, were you aware that any support options were available?
  - *Follow-up question:* How did you then find support *– e.g*
  - *Prompts:* e.g. through the internet? By meeting other caregivers?
- How do your current support needs compare with your support needs in the past?
  - I.e. Can you distinguish your current needs from anything that might have occurred in the past but you have now moved on from – did you struggle with anything previously?
- How easy has it been to access help or support when you’ve needed it?
  - *Prompt:* Does/did it take a long time to get help, or was/is it a relatively quick and simple process?
- What help and support has been most beneficial for you in your caregiving role?
  - *Follow up*: What’s a good example or positive experience you’ve had with support services?
  - What was it about this particular [service/intervention etc] that made such a difference?
- Which aspects of supporting your family member would you most like extra help with?
  - *Prompt:* Are there particularly stressful aspects of [caregiving] that additional support might help you to cope with?
  - What form would this support take?
- How do you feel support for caregivers of people with visual impairment could be improved?
- Would you consider yourself a “caregiver” or “carer”? Are those terms you would identify with? Is there a better term to describe your role?
- In your view, what do you think makes [caregiving for/supporting] someone with a visual impairment different to [caring for/supporting] people with other health conditions or disabilities?
  - *Prompt:* What particular challenges does visual impairment pose for them… and for you as the person they might turn to for help?
- Where do you feel the gaps are in the current support system for [caregivers/friends/relatives/parents/supporters] of visually impaired people in the UK?
- What additional support would you find helpful going forward, for you specifically?
- Is there anything else you feel I should have asked, or anything you would like to add or discuss further?
