# Supplementary material for: An exploratory study on support for caregivers of people with vision impairment in the UK
Source: Ophthalmic Physiol Opt. 2022 Apr 13;42(4):858–71. doi: 10.1111/opo.12989 (PMC9320821; doi:10.1111/opo.12989)
Supplement: Supplementary file 3 — Appendix S3 [file OPO-42-858-s001.docx]

## Appendix 3: Table showing complete service use and demographic information of participants, with CSQ scores by group

| **Variable** | **N (= %)** | **CSQ-8 score:** Scores can range from minimum 8 to maximum 32  **Mean (SD)** | ***P-*value** |
| --- | --- | --- | --- |
| All participants | 100 | 21.6 (7.2) | N/A |
| From which kinds of organisation have you received support as a person supporting a visually impaired person, if any?^†^  Healthcare services  Charities  Social services  None | 41  61  22  13 | 23.9 (5.1)  24.6 (4.6)  23.8 (5.2)  9.8 (2.5) | 0.02  *<0.001  0.20  *<0.001 |
| Have you been given the details of a person who you can contact if you have any questions, worries or concerns about visual impairment or caring for someone with visual impairment? |  |  | *0.003 |
| Yes | 39 | 24.4 (6.5) |  |
| No  *Did not respond* | 59  *2* | 19.7 (7.2)  *22.0 (2.8)* |  |
|  |  |  |  |
| Have your needs as a caregiver been assessed by any health professional in the last 12 months?  Yes  No  *Did not respond* | 7  91  *2* | 22.6 (8.6)  21.5 (7.2)  *22.0 (2.8)* | 0.91 |
| When decisions are made about the care or treatment of the person you support, do you feel that your views and needs are taken into account?  Not at all  Hardly ever  Sometimes  Often  Always  *Did not respond* | 19  11  24  24  10  *12* | 17.7 (8.0)  17.5 (7.3)  22.0 (6.9)  24.1 (5.5)  25.8 (5.2)  *22.4 (7.5)* | *0.01 |
| Overall, how would you rate the level of support which you have received from the health services in the last 12 months?  Not applicable  Not supported at all  Some support  Enough support  Very well supported  *Did not respond* | 10  32  23  13  10  *12* | 25.1 (5.2)  18.1 (7.9)  20.0 (5.7)  24.6 (5.6)  28.1 (3.5)  *22.4 (7.5)* | *<0.001 |
| Do you feel the support you receive could be improved?  Not at all  A little  Somewhat  Quite a lot  Yes, a lot  *Did not respond* | 11  14  20  18  25  *12* | 26.8 (5.9)  25.4 (5.8)  23.1 (5.2)  20.9 (6.0)  16.1 (7.4)  *22.4 (7.5)* | *<0.001 |
| Age, y  18 – 24  25 – 34  35 – 44  45 – 54  55 – 64  65 – 74  75 – 84  Over 85  *Did not respond* | 1  5  19  28  14  14  4  1  14 | 32.0  22.0 (6.9)  22.3 (6.8)  21.5 (7.0)  22.6 (7.1)  19.0 (7.5)  21.8 (9.0)  30.0  20.9 (8.0) | 0.60 |
| Ethnicity  Asian or Asian British  White British  White Other  Would rather not say  *Did not respond* | 2  76  5  3  14 | 17.0 (7.1)  21.9 (7.1)  23.0 (8.1)  17.3 (4.9)  20.9 (8.0) | 0.55 |
| Gender  Female  Male  *Did not respond* | 67  19  14 | 22.1 (6.8)  20.5 (8.1)  20.9 (8.0) | 0.80 |
| Marital status  Divorced  Living with partner  Married  Single  Widowed  Would rather not say  *Did not respond* | 4  7  70  3  1  1  14 | 22.8 (4.1)  23.0 (5.9)  21.9 (7.3)  14.7 (7.0)  23.0  15.0  20.9 (8.0) | 0.63 |
| Religion  Buddhist  Christian  Hindu  Jewish  Muslim  No religion  Would rather not say  *Did not respond* | 1  50  1  2  1  28  3  14 | 27.0  22.9 (6.2)  12.0  18.0 (7.1)  22.0  21.0 (8.2)  12.3 (3.8)  20.9 (8.0) | 0.27 |
| Do you consider English to be your first language?  Yes  No  *Did not respond* | 85  1  14 | 21.6 (7.1)  29.0  20.9 (8.0) | 0.50 |
| Highest education level achieved to date  No formal qualifications  GCSE (or equivalent) [exams taken at 16 years of age]  A-Level (or equivalent) [exams taken at 18 years of age, at end of high school]  Undergraduate degree  Postgraduate degree  Other professional training  Would rather not say  *Did not respond* | 2  14  16  32  14  5  3  14 | 29.5 (3.5)  20.9 (8.2)  19.0 (7.7)  21.9 (7.3)  24.4 (5.1)  24.4 (1.9)  15.7 (2.1)  20.9 (8.0) | 0.18 |
| Do you currently suffer from any chronic (long-term) health condition(s)?  Yes  No  Would rather not say  *Did not respond* | 26  57  3  14 | 21.0 (7.3)  22.3 (7.0)  17.3 (6.7)  20.9 (8.0) | 0.55 |
| Relationship: Is the visually impaired person you support/care for your…?  Child  Parent  Partner/Spouse  Sibling  Other  *Did not respond* | 41  15  26  1  3  14 | 22.7 (6.3)  23.9 (7.1)  18.9 (7.9)  23.0  21.3 (7.0)  20.9 (8.0) | 0.38 |
| Level of VI: Does the person you support/care for have…?  Mild visual impairment  Moderate visual impairment  Severe visual impairment  I do not know  *Did not respond* | 7  32  46  1  14 | 25.6 (3.0)  22.4 (6.0)  20.7 (8.1)  19.0  20.9 (8.0) | 0.64 |
| Legal certification: Is the person you support/care for…?^ǂ^  Legally certified as severely sight impaired (“blind”)  Legally certified as sight impaired (“partially sighted”)  Not certified  I do not know  *Did not respond* | 33  37  14  2  14 | 21.3 (7.8)  21.4 (7.0)  23.6 (5.8)  20.5 (9.2)  20.9 (8.0) | 0.89 |
| Does the person you support/care for have another chronic (long-term) condition as well as their visual impairment?  Yes  No  I would rather not say  *Did not respond* | 30  53  1  16 | 20.7 (6.3)  22.8 (7.2)  8.0  20.1 (8.1) | 0.09 |
| Does the person you support/care for have any of the following conditions that are linked to visual impairment (please tick all that apply)?^§^  Age-related macular degeneration (AMD)  Cataracts  Cerebral visual impairment  Charles Bonnet Syndrome  Diabetic Retinopathy  Glaucoma  Neurological disease (e.g. visual impairment after stroke or trauma)  Nystagmus  Rare inherited eye diseases (e.g. Retinitis pigmentosa, Leber congenital amaurosis, Stargardt disease)  Other | 18  18  5  4  2  8  3  23  22  14 | 24.1 (6.0)  19.9 (7.5)  19.0 (7.9)  23.8 (6.7)  21.5 (0.7)  19.3 (7.8)  14.7 (10.7)  23.3 (6.9)  18.3 (7.5)  19.9 (7.2) | 0.12  0.28  0.42  0.47  0.66  0.31  0.20  0.17  0.02  0.28 |

^†^ For this question, participants could select more than one response, i.e. all those that applied. Mann-Whitney U tests were carried out to see if CSQ scores were significantly different among those who reported receiving support from the relevant sector, versus those who did not report any support from that sector. *P-*values were considered significant at the Bonferroni-corrected level of 0.0125 (based on 0.05 divided by 4, as 4 tests were carried out). Thus caregivers who reported receiving support from healthcare services tended to have higher CSQ-8 scores than those who did not report receiving such support, although this did not reach statistical significance (as *P* = 0.02).

^ǂ^ These categories are based on how vision impairment is certified in the UK. More detail is available here: <https://www.rnib.org.uk/eye-health/registering-your-sight-loss/criteria-certification>

^§^ For this question, participants could select more than one response, i.e. all those that applied. Mann-Whitney U tests were carried out to see if CSQ scores were significantly different among those with the disease versus those without. *P-*values were considered significant at the Bonferroni-corrected level of 0.005 (based on 0.05 divided by 10, as 10 tests were carried out). Thus while those who were providing support for someone with rare inherited eye diseases tended to have lower CSQ-8 scores than those without, this did not reach statistical significance (as *P* = 0.02).
