# Supplementary material for: An exploratory study on support for caregivers of people with vision impairment in the UK
Source: Ophthalmic Physiol Opt. 2022 Apr 13;42(4):858–71. doi: 10.1111/opo.12989 (PMC9320821; doi:10.1111/opo.12989)
Supplement: Supplementary file 4 — Appendix S4 [file OPO-42-858-s004.docx]

Appendix 4: Extended qualitative analysis and data extracts

NB. Information in brackets refers to the person with visual impairment (VI), in terms of who the person with VI represents for the participating caregiver and the severity of their VI. For example, if a participant is quoted as (Child, Mild VI), this means that the quoted participant is the parent/caregiver of a child with mild VI.

# Theme 1: A limited support infrastructure for caregivers

| **Description** | **Illustrative quotation(s) – with numbers referred to in article text** |
| --- | --- |
| Many participants stated that either they had never thought about the possibility of support for themselves, or that nobody had ever thought that they might be in need of support. | 1. *More support needed at initial diagnosis* (**Child, mild VI**). 2. *Nothing has ever been said to me about where I can go for any support myself* (**Child, severe VI**). 3. *I have never been asked if I needed any support in caring for my daughter, maybe because she is not a minor* (**Child, severe VI**). 4. *People just kind of focus on the person with the sight loss. I don't think people really think about people that are supporting somebody with sight loss* (**Spouse/partner, severe VI**). 5. *I feel that there is nothing out there to help us at all* (**Child, severe VI**). 6. *In terms of direct support for carers, I haven't seen any in the landscape…Carers do get pretty desperate. We would love some help, for ourselves* (**Parent, severe VI**). |
| Some participants struggled to respond to the survey because they had not received any support which they could subsequently evaluate. | 1. *These questions are hard to answer because it is actually difficult to identify any specific help that I have received as a carer* (**Parent, moderate VI**)*.* 2. *Initially on the questionnaire there was no opportunity to say I was getting no help which made answering some questions very difficult. I do what I do for my wife because I need to. It would help if I knew what was available.* (**Spouse/partner, severe VI**) 3. *Your questions ask about "the help we get"… well we don't get any... but that is because we don't ask for any* (**Parent, moderate VI**) 4. *I haven't received any help because I haven't really needed it. But if I did need help I wouldn't know where to get it* (**Spouse/partner, moderate VI**). 5. *This is the part that really surprised me about the whole survey. It had never occurred to me that there might be support [for caregivers]… That's the funny thing about it* (**Parent, moderate VI).** |
| The lack of support for caregivers was often considered to be closely linked to the lack of support for the person with VI. | 1. *My husband has an aggressive inherited eye condition.... I have never been offered any direct support or advice about how best to care for a person with visual impairment. (My husband has also never been offered support and has always had to find it himself.) The non-clinical support of people with visual impairment, and their carers, is hugely neglected in my view* (**Spouse, moderate VI**). 2. *Support is massively lacking across the board for the both of us* (**Child, severe VI**). 3. *I have been shocked at how little help and support is available for VIPs, let alone their full-time carer/s* (**Parent, severe VI**). |
| Some participants believed support could be offered earlier and more proactively before circumstances became overwhelming, especially when VI occurred suddenly. Misdiagnoses, delays with diagnosis, or poorly delivered advice or communication from health professionals were significant sources of distress, particularly for many parents/caregivers of children with VI. | 1. *Nobody offered us anything until I hit rock-bottom* (**Spouse/partner, severe VI**). 2. *You need more support at the beginning, it's a minefield, you've never had that in your life before… If you're thrown in the deep end, where do you go for help for this? How do you manage to deal with this?... The longer you help somebody with sight loss, the more you get used to it I suppose* (**Spouse/partner, severe VI**). 3. *There was no real support, just being told that your child has a disability in the first place - you know it's kind of: here's the diagnosis, there's the door. And that doesn't really help* (**Child, moderate VI**). 4. *So then they suggested registering her as sight impaired... And they said, 'Oh, you know, she probably won't be able to drive', and that was it. We left the room, no leaflets, no information, nothing at all* (**Child, severe VI**). 5. *I'd like to have walked out of that consultant's office with some information, websites, any kind of place I could go and get some information. I would have liked to have been assured that he's ok, quite normal, not brain damaged, will not affect anything, just to love this child as a child. I'd have liked the consultant to say, right this is what we're going to do, we're going to do some more investigations and try to understand why and what it is, and if you go to your local education service you'll be able to access this, and if you'd like to talk about it you could go to these people, and you'd be given a load of places to go and source information from* (**Child, moderate VI**). |
| Responsibility falls to the caregiver to proactively seek support, or to learn how to cope through trial and error. Some participants described coming across relevant guidance and information only by chance. | 1. *All too often it is the parent that has to guess the problem and then seek out the cause* (**Child, moderate VI**). 2. *I have had to contact everybody... none of them have really contacted me... they're not massively proactive* (**Child, moderate VI**). 3. *There are posters up in the Eye Hospital about going to speak to someone but it is incumbent on us to go find them* (**Spouse/partner, severe VI**). 4. *Although the extent of my mother's sight loss is known to the GP, I had to push to get an appointment for her to be assessed as partially sighted to enable her to access help from the Low Vision Clinic and the Sensory Impairment Team etc* (**Parent, moderate VI**). 5. *I only picked them [the leaflets] up from the ophthalmologist's sitting room where we waiting... and then eventually I happened to be reading them because I was sat there doing nothing, and then we found out the information about what was wrong with [child] - in more detail than we did from going to the ophthalmologist* (**Child, severe VI**). 6. *Well, there are leaflets around, and you're just sort of expected to find the leaflets* (**Parent, severe VI**)*.* 7. *They [health services] don't even tell you at that stage that you might be able to get assistance, or where you might get it from. You only sort of gradually learn these things...* (**Parent, moderate VI**). 8. *If you're not proactive, if you wait, nothing happens… I reach out* (**Spouse/partner, moderate VI**). |
| Participants described wide variation in how far diagnosing clinicians refer or signpost to further support. Sometimes, support was only ‘unlocked’ through a chance encounter with an individual. | 1. *Unless the hospital had been proactively referring, I wouldn't have known they existed* (**Child, moderate VI**). 2. *I was just going to the health visitor anyway... and when [child] got the diagnosis, she just stepped up the support I suppose... she would come and visit us after a hospital visit and make sure we knew what we were doing. We were very lucky I think, and very well supported - I don't think it's always like that but for us that was good* (**Child, moderate VI**) 3. *The one person we got a stonking amount of support from was the health visitor... such a lovely woman, and we kept in touch with her. She was absolutely superb… Having that person was great. She understood right from the moment [child] was born and what we went through... But anybody else, nothing - we weren't referred to anybody.* (**Child, severe VI**). 4. *We had a very good specialist locally who we knew we could contact if we needed her* (**Child, moderate VI**). 5. *There is support available for carers around the district if you know where to get it. But knowing where to get it is the basic problem…Without direct contact with our neighbour it is doubtful that we would have known about this support* (**Spouse/partner, severe VI**). |
| Participants discussed the pressure to be coping or ‘managing’, and so it can be difficult to ask for help or support. | 1. *For a lot of people, it's really difficult to ask for help - that's probably one of mine and my husband's massive downfalls is knowing how to ask, because you just don't want to seem like you can't cope... If it was one of my friends, I'd tell them go to this place, go to that place, I'd go and do the research for them and give them points of contact, if it was one of them. But for yourself, you just think, ‘Mmmm, no, I can manage’* (**Spouse/partner, severe VI).** 2. *Pride is a barrier to getting support* (**Spouse/partner, severe VI**). 3. *One thing we found out, especially on the last holiday we went on, is that if you actually ask for help, there's a lot more out there that we hadn't really experienced before, you know* (**Spouse/partner, moderate VI**). |
| A small minority of participants - especially when the onset of VI had been gradual – thought that no additional support was required. | 1. *Nothing needed… my husband has no sight now but we manage perfectly well without outside help. We go away on our own several times a year and keep very active* (**Spouse/partner, severe VI**). 2. *We hadn't been offered any support, but I know it's there. I suppose because we've been coping well anyway, neither of us felt that we needed support, or I needed support… Because it has been so slow, and we were told that it would probably be slow, we've coped gradually over time quite well with it, because it's been such a slow onset* (**Spouse/partner, severe VI**). 3. *Her sight loss has been slow allowing us both to adapt… Life is good! We know where to get support if needed* (**Spouse/partner, severe VI**). 4. *I would say I haven't actually needed any support. You know, I've been quite happy just to use common sense and get on with it* (**Spouse/partner, severe VI**). |
| There was awareness from participants that their needs for support could change significantly over time or if there were a sudden shift in circumstances. Caregivers’ own health or disability, for instance, could affect the level of support that might be required. | 1. *I now realise that the disease takes many forms and twists and turns in its journey and everyone is different - and that in the future we may need more help and support - which thankfully we have not yet needed. I am sure I will be able to access support when we get there* (**Spouse/partner, moderate VI**). 2. *I think I know exactly what I've been doing, and I've been doing it a long time... if she developed a physical condition as well, then that would be a totally different kettle of fish, and I might need some support then* (**Spouse/partner, severe VI**). 3. *I'm classed as disabled so sometimes it's hard to kind of keep on top of things if you're trying to keep on top of your own stuff as well* (**Spouse/partner, severe VI**). |
| Information is not necessarily the same as support. Support and information to be a ‘better’ caregiver, for example in the form of advice from a low vision clinic or a sight loss charity, was considered very different from support for the caregiver in their own right. | 1. *I have received very little support for ME as a caregiver. I have received answers to my requests for information about how to best support my young person - but this is not quite the same as receiving support for me. If I proactively look for support to help me be a better carer I can find it - but I cannot think of any organisation who has offered me support solely because I am a carer of a VI young person* (**Child, moderate VI**)*.* 2. *Availability of the information is critical…The bit with the access to information, that was an obvious gap in the early stages of [child]s] life* (**Child, severe VI**). 3. *Information is the only support that I have ever received, which is important. But in terms of actually how to survive as a human, I've just had to do that myself… I have found information useful, but I'm fortunate that I'm well educated, English is my first language. I'm hugely better placed than a lot of people to understand the information that they're giving, and I'm able to question and make sure that I understand what it is and then I can go away and act on it or read articles or whatever it is. For some people, even just understanding the information would be much more difficult* (**Child, moderate VI**)*.* |
| Information has to be tailored, taking into account the differing demographics and circumstances of caregivers. | 1. *My concerns are for carers of ethnic minorities whose first language is not English - where do they get any support or advice for their roles? Most of these carers do not know where to get support from. They generally do the caring naturally, and require help and support and to know where to get what support from* (**Parent, severe VI**). 2. *Being given care advice based on ‘information for retired sufferers’ for a wife in her 30's was, frankly, insulting!* (**Spouse/partner, severe VI**) 3. *It may not be good to give all the information at the beginning and it could be that I have not had support because I have not needed or asked for it yet* (**Spouse/partner, moderate VI**). 4. *For us people who sit there and are just there to support, I’m not sure that there’s a great deal of focused information for us. You know, I don’t remember ever being handed a leaflet and said, ‘Read that’. I’m the kind of person who picks it up and reads it. But if I’m not that kind of person, would I have done it?... It just gives me the impression that family members don't get targeted with information. It's there... but you have to go and look for it… The other thing is that perhaps people are told, but the first thing they've heard is, 'You're going to have to have an injection'. Now how much of the rest of the conversation are you going to remember?* (**Spouse/partner, moderate VI**) |
| Access to reliable information can be challenging. There were also concerns from some participants that information is mainly online, and so may exclude those with limited computer skills or access, or may not be trustworthy. | 1. *Unless you're into computing, you're not going to see the information* (**Spouse/partner, severe VI**). 2. *I don't think we're lacking information, but you never know with the internet what you're reading… Sometimes you don't know the providence of the information you're reading* (**Parent, moderate VI**). |

# Theme 2: Disparities and complications accessing support

| **Description** | **Illustrative quotation(s)** |
| --- | --- |
| Finding the right service could be a key issue for caregivers. Some participating caregivers felt it was unlikely that they would ever ultimately find the right support service; evoking a feeling of being passively passed between agencies. It can also be challenging for caregivers to know which service or guidance is right for them if they are seeking help. | 1. *Everyone seems to refer me on to someone else* (**Parent, severe VI**). 2. *[Our area] has a tremendous amount of different support services. The problem here is, there are so many, choosing the one which is right for you is quite difficult* (**Spouse/partner, severe VI**). 3. *A lot of the guidance that you get, sometimes there's too much of it as well, it's quite strange - because some of the things you're not eligible for anyway. So really you almost need some kind of flowchart to follow, to work out which kinds of things you're going to get and which you're not. Because you waste a lot of time working out whether this would be right for you or not* (**Parent, moderate VI**). |
| Parents often saw the support system for their child as overstretched. They felt unsure of what support was materially available, as well as their due/legal entitlement to support, which led to them feeling they could be missing out on access to a crucial service. The need for their child to access support often felt particularly urgent, due to fears that delays could significantly affect their child’s development. This in turn could create significant stress for the parents. Sometimes support for children with VI had eventually fallen into place, following long waits and delays. | 1. *[It would help] knowing what support my VI child can get, should get by law, how to access them. You don't know what you don't know...it's all a hidden maze* (**Child, severe VI**). 2. *The most stressful part of my role is trying to tie everything together, find out what support she should be getting. Are we missing anything?...As much as I try and do my research and I try and read up on things and I try and understand what's there, I don't know if I've missed something* (**Child, severe VI**). 3. *There was a visually impaired support unit... that came [to the school] once I think… but they were so overwhelmed with people with serious visual impairments... that they were just overstretched* (**Child, mild VI**). 4. *[My child] is absolutely living her dreams, and finally is at a VI college who have the facilities to ensure things are in place to ensure she can learn independently and with the right adaptive technology. I guess it’s just a shame that this has taken our local authority almost until she’s turned 16 to get it right!* (**Child, moderate VI**). 5. *The lack of support through primary school affected him greatly until just recently. Secondary school and the Council have been brilliant… and the Guide Dogs have provided an enormous amount of support too. If we had been given guidance and support from age 4 through to 11 years old, I believe my son would have had more confidence in his abilities and as a person during that time* (**Child, moderate VI**). |
| Some participants had hoped that certification or registration as visually impaired might be the start of a more formalised support pathway, which had not yet materialised. | 1. *When the registration [as visually impaired] was done, it wasn't terribly clear what would happen next... but finally with some prompting, the local authority have now acknowledged that they will be contacting us. And once they do that, about six months will have passed in between. So it's not straightforward, and it's also not obvious who should take the initiative - whether they're going to contact you or whether you should have to remind them... You're left wondering what should be happening now* (**Parent, moderate VI**). 2. *So we sort of said, 'Look, if you think she is classified as significantly visually impaired, please can we register her as that, because we're struggling to get help, and if we can register her as that, then, you know, that might give us extra help’. So they did that, and then we left, and there's been no support since then - it's been quiet* (**Child, severe VI**). |
| Participants were aware of disparities in support available from different local authorities, hospitals and schools. Moving between local authorities often led to a change in the standard of support received, as well as problems in the actual transition. However, moving could also result in improvements in support service access and quality. | 1. *My friend... his sons have an identical condition to my son, and they've had weekly mobility sessions for years and my son has never had any…The disparity… the difference between the two, for the same condition… Actually on paper, [my child's] condition is more severe and yet he has significantly less support, which is quite poor really, if you look at it that way* (**Child, moderate VI**). 2. *Through some paperwork going to the wrong place… something went wrong. So all the support we've requested has always been from us asking [the council] directly, and my daughter didn't have QTVI [Qualified Teacher of the Visually Impaired] support for six months because we were between council areas, and so we were dropped from one council list and weren't put on another one, and we were waiting to hear back from different people and noone ever got back to us, and in the end it was six months where she didn't receive any help at all… And this is at a crucial age* (**Child, severe VI**). 3. *We've moved from one council to another. People have rung me and have said they've sent me an email, but we haven't actually spoken to someone from that team yet. Whereas with the other person in the other council, we had a direct number and we could get through to that person straight away. So sometimes it's quite frustrating trying to get through to the right person* (**Spouse/partner, severe VI**). 4. *Since moving… we have had a lot of support from the local Macular Society group. This has opened up to us the care and support services that are available. This has proved to be very beneficial* (**Spouse/partner, severe VI**). 5. *Last time, the family liaison officer was talking to us, and [child] was registered but we had moved area… and I didn't know whether the registration automatically went to the new local authority. So she told me what to do to get all that checked - it hadn't, so I've now made sure they've got all the relevant information at the local authority... So it's really helpful, just to have that holistic overview of what we should be doing to make sure [child] gets the best* (**Child, moderate VI**). 6. *And actually since I filled the survey in, I've gone online and checked, and our council are absolutely brilliant. You look on there and the support for carers, the pathways that they can show you to find support... I was quite surprised by just how much help there is out there for carers… And it’s really easy to navigate through* (**Spouse/partner, severe VI**) 7. *We were lucky because not all cities in the country offer [support with Personal Independence Payment claims], but luckily they did here* (**Child, severe VI**) |
| Frustration at the “postcode lottery”, and the short-termist lack of support for PVI and caregivers, which can result in costs later down the line. | 1. *The government don't look at it now, but if you tally up all the years they put into resources, to have something in place like a safe space for people to talk, maybe in local authorities, maybe that you could join, once a year you had a... reunion. Just one thing that if you needed to and were able to, you could go. But the main issue with this is cost. I would say to the government, they need to put more money into, and less pressure onto the caring person, so that you have time yourself, you have places to go yourself, and that you have the correct and up-to-date information. Because it takes a long time to pick up the phone, and you pick up the phone and nobody's there anymore, and they haven't updated the website... It's all to do with support, money, local authorities and the postcode lottery* (**Spouse/partner, severe VI**) 2. *You could use the argument that better support early on saves money later* (**Child, mild VI**) |
| Participants generally expressed more favourable views of charity than statutory (i.e. NHS, local authority) support. Some participants discussed how the voluntary sector is increasingly having to provide support that should be available through NHS or social services. | 1. *That's why when we're asked, how good has support been, it's been very very good from the voluntary sector, but from the NHS, and from the local Council and whoever else is meant to be providing help, it's been either very poor or non existent… But with [a national sight charity], there were no barriers to getting help... I was surprised at how proactive they were and how helpful they were, and it really is welcome as we're all extremely busy day to day and we're trying to do the best for our daughter... and they really went above and beyond*” (**Child, severe VI**) 2. *There’s a total reliance on the voluntary sector, for starters. There's a total reliance on people knowing those voluntary organisations are there. And if you suddenly become visually impaired, who tells you about them? I wouldn't be confident that the hospital would tell you... They might do, you might be put in touch with the liaison officer... but I wouldn't necessarily bank on it, the way things are. So you know, a more proactive approach to offering support and publicising support, making people aware of any support... just more out there.* (**Parent, severe VI**) 3. *With cutbacks, there's far less available than 10 years ago. And a lot of it is being picked up by volunteers setting up their own groups* (**Spouse/partner, severe VI**) |
| Charities may not cater to caregivers living in rural areas, or working full-time. Charities operating at the local level may provide more tangible support than national charities. | 1. *With a big charity… they're mainly city focused, they have lots of facilities for disabled people, regardless of what the disability is, and people are more aware of it. Whereas if you're more rural, out-in-the-sticks kind of thing, the facilities aren't there, or people are less aware of 'situations', so to speak…The national charities are so big... you know, it's almost like it's too big to contact, cos it's just like, well, whereabouts in the charity, who do you contact, which section do you contact*? (**Spouse/partner, severe VI**) 2. *The charity is only open three days a week and these are during office hours. I work full time Monday to Friday so cannot usually get to see or talk to anyone* (**Parent, moderate VI**). |
| Caregivers advocated a single, consistent, informed point-of-contact, who might be able to provide advice or at least to signpost directly to the right service. They might not have all the answers but ultimately might be the key link to unlocking and accessing appropriate support. Participants suggested regular contact with a person akin to an ECLO, support/social worker, health visitor or specialist nurse. | 1. *Someone you could sit and talk to about what it actually means in terms of what they can see, what they can't see, and what the problems might be* (**Child, mild VI**). 2. *Someone to phone once in a blue moon, just saying, 'How's things now, do you need any more support?' Because I think a lot of times it can be isolating, and you don't know where to turn... A bit like the support from the Stroke Association, where they ring every so often, if you want or need it, someone just kind of checks and sees how you are* (**Spouse/partner, severe VI**). 3. *A regular support worker assigned to the carer and informed on the needs of the visually impaired* (**Child, severe VI**). 4. *You need a person to call… Here, we had a specialist diabetic nurse. And that was wonderful. Because once we got that, you always went to the right place. Otherwise you got passed from pillar to post, and nobody ever knew the answer to anything. But these specialist health people were of great use... That's what you need* (**Sibling, moderate VI**)*.* 5. *In the way that you get a health visitor… even if it was just once a month that somebody came round to just check that the primary caregiver of a disabled child is just, kind of, OK. To know that there was somebody who was there for me, and not just for [child]. I never had that* (**Child, moderate VI**). 6. *Just someone who can be a point of contact, who, if I've got any concerns or worries... basically, it might not be that I'd need to actually use them, but just to have that point of contact to say, ‘This is what's going on at the moment, how do I handle this situation?’… Just to give that bit of support when you need it really, because it's not that you do always need support, it's just nice to know it's there when you do need it, because invariably there will be points where you do* (**Spouse/partner, severe VI**). 7. *Someone with the local knowledge to know who to ask in the Council, who to ask in the charities... and maybe not do it for us, but to point us in the right direction. Put the onus on us, but just point out that something's gone wrong. It's not that I'm expecting someone to do it for me; it's just expecting someone to provide that single point of contact for all the services that [our child] will be needing* (**Child, severe VI**). 8. *In an ideal world, you need a personal social worker who turns up or is on the phone, available to you, who knows you. And you could say 'Look, all's fine, I haven't got any problems'. Or they would be proactive and ring you up and say, 'How are you today? How's the dog?', or whatever they want to talk about to get you talking. And then you could get some assistance. But it's unlikely to happen in this financially constrained health service* (**Spouse/partner, moderate VI**). 9. *Someone to call… you might not get a result and the outcome you need, but you might get a link to it, the start of the process… You need a continuous programme, so that if someone leaves that post, someone steps in* (**Spouse/partner, severe VI**). |

# Theme 3: Support for practical day-to-day challenges

| **Description** | **Illustrative quotation(s)** |
| --- | --- |
| The financial implications of caregiving were concerns for many participants. For example, participants noted the high costs of some of the visual aids and assistive technologies the PVI might require or benefit from. There were also sometimes prohibitive costs associated with accessing available statutory or voluntary services, such as personal assistance or transport. | 1. *People could access cheaper ways of buying very strong glasses* (**Child, severe VI**). 2. *These glasses are life changing for her. She more than deserves them. I must hold on to this fact despite the financial cost* (**Spouse/partner, severe VI**). 3. *[I need]* *proper financial support for assisting technology for my mother* (**Parent, severe VI**). 4. *[I need] easily accessible information on the financial and additional support that can be given to a carer* (**Spouse/partner, severe VI**). 5. *[My child with VI] has a social worker but unless we pay for companion/assistance (which we cannot afford) then he cannot access this service* (**Child, severe VI**). 6. *The cost to get there [to peer support events/meetings], the cost to stay over, and the cost to go… A lot of people out there haven't got the resources* (**Spouse/partner, severe VI**)*.* |
| Low vision aids: Participants discussed access to assistive technologies and low vision aids as a potentially valuable form of practical support. One participant highlighted the importance of affordable visual aids, and the possibility of trying out different options. | 1. *[I would like] more directed support (e.g. parent groups or seminars) about my child making the best use of technology* (**Child, moderate VI**)*.* 2. *It would be useful to know places that are knowledgeable about certain apps that will help? Or equipment, that may make our lives a bit easier* (**Spouse/partner, severe VI**)*.* 3. *Support teams are not fully aware (or have the capacity) of nature of help required. Instead of establishing the nature of support to be provided, they look at giving support they can give which often is not what people with visual impairment require. Primarily it is critical that support service teams are kept abreast with technologies, and are well informed of solutions available* (**Child, moderate VI**). 4. *There is a place locally where you can go locally and examine the various aids and so forth... that's the sort of thing that I think is most useful, actually being able to go to a place, try some things out and take something away and use it, and if necessary go back and swap it for something else… If equipment was available on loan, that would be more interesting. It's not generally interesting to know you can buy a piece of kit for £600 to £1000 and you might find that you can't use it, after you've purchased it. That's really the sort of issue that you have* (**Parent, moderate VI**). |
| Benefit applications: Participants referred to the difficulty filling in forms with or on behalf of the PVI to apply for DLA [Disability Living Allowance] (and its replacement, PIP [Personal Independence Payment]) which were often found to be long, burdensome and complex. Some participants had spent significant time embroiled in legal proceedings and awaiting decisions in order to access financial support. More financial support upfront could end up saving funds in the long-term, by providing support before both the caregiver and PVI’s problems become too acute. Help with the benefit application forms could be a useful form of support for caregivers. | 1. *I have done this role for 15 years but only got carer’s allowance when she moved from DLA to PIP* (**Spouse/partner, severe VI**). 2. *People struggle with the disability living allowance application forms… that's a bit hit-and-miss. You've got to go digging around… we weren't pointed in any direction, it was a Google search and then writing off emails to lots of different people, asking can you help us with this?* (**Child, moderate VI**). 3. *There's no help on hand… if you can't fill out forms, there's no-one around to say 'I'll help you', no-one comes to ask if you're having a problem* (**Spouse/partner, moderate VI**). 4. *Sixteen years on and I'm still a carer that is battle wounded from trying to change the law for others to get full benefit entitlements. The Department of Work and Pensions [need] to change their ruling on 'total blindness' for benefits. My wife has 1% field of vision and, therefore, misses out on full high rate despite us challenging this rule in the courts* (**Spouse/partner, severe VI**). 5. *Social Services are currently assessing financial support for caring. If the result is draconian then the family will implode* (**Child, severe VI**). 6. *[The PIP application form] is soul-destroying, because you’re focusing on all the negatives* (**Child, severe VI**). 7. *[I need support] attending the ESA [Employment and Support Allowance] interview* (**Child, severe VI**). 8. *The government should tally up all the years they put into resources. I know funding is horrific out there. But if they have a breakdown, the family, that cost... surely it's better to try and hit it earlier on than wait for the process of them needing medication for the rest of their life, counselling for the rest of their life* (**Spouse/partner, severe VI**). 9. *This chap came about 2 or 3 times and spent a total of about six hours with her, and he went through the form with us - he was so helpful. And that's probably the most help we've had, he was fantastic - and he came to the assessment with her and with myself, and she was awarded the PIP and she's not got to be reassessed for ten years, so thank goodness for that* (**Child, severe VI**)*.* |
| Employment: Participants described the intense stress of trying to balance employment and caring responsibilities, and feeling torn between employment and caregiving. | 1. *[We need] the ability to claim carers allowance and work full time* (**Spouse/partner, severe VI**). 2. *[I need] support to hold down full time employment as well as spend time caring* (**Child, moderate VI**). 3. *I have given up work to look after my child which I wouldn't have done if they were not VI. So my world/life has changed massively, but no support has ever been offered to me* (**Child, severe VI**). 4. *It is assumed that I will always be there. And because I work, I cannot get Carer's Allowance which may help me pay someone else to do some of the things I have to do. But I do work and cannot always be available when needed due to this. It stresses me out… But what do I do, become voluntarily unemployed and the BA [benefits adviser] on my back to find work? No win situation* (**Spouse/partner, severe VI**)*.* 5. *I cannot get any help financially as I work full time. I have to take a day’s leave to attend [child with VI’s] weekly appointments at [Eye Hospital]. I have to rely on my parents to look after him whilst I go shopping which is not ideal. I feel that there is nothing out there to help us at all. I cannot afford to give up work as I still have a mortgage* (**Child, severe VI**). |
| Transport: Caregivers will often accompany the PVI to appointments, or may have issues travelling to attend carer support services. Several participants felt they would be helped by a blue badge for the person they were supporting. One potential solution mentioned by several participants separately was a kind of “befriending” or “buddy” system, whereby a known person in the local area could be counted upon to drive the person with VI to appointments on a semi-regular basis, thus supporting caregivers who cannot drive. | 1. *Carers groups are not accessible without transport* (**Spouse/partner, severe VI**). 2. *[It would help to have] a Blue Badge for my wife who is partially sighted with macular and is unsteady when walking* (**Spouse/partner, severe VI**). 3. *The blue badge helps immensely when we go for appointments* (**Child, severe VI**). 4. *It could be a volunteer or buddy who would become a friend over the years... Certainly it wouldn't be that onerous a job - it could be quite a pleasurable task... if it got to the stage where we couldn't drive or something, just the fact that you could ring up… If it was somebody who was already like a buddy, it's perhaps the sort of thing you could ask them if they'd be prepared to do it, even if you had to pay this person - it would be great to know you could ring up this person rather than a taxi company...* (**Child, severe VI**). |
| Mobility: A large number of participants spoke of the need for more mobility support, especially in terms of training with how to guide or support the PVI with getting around. | 1. *Mobility is really difficult to get for somebody who's got difficulties with vision in the dark. Everyone works 9-5 don't they pretty much, but that's not when [child's] issues are, and if I want him to be independent, he needs to know how to get around at night* (**Child, moderate VI**). 2. *[It would help to have] training on guiding a VI person* (**Spouse/partner, severe VI**). 3. *He really needs to use the white cane now. And [it would help] if there were people to ask about how they got around that with their partners, or how people dealt with that situation. Because… he really needs to use it, but I also understand why he doesn't want to use it. So it's like, how do you deal with that situation? How do you persuade someone, and how could I guide him better?* (**Spouse/partner, severe VI**). 4. *[It would help to have] the removal or ban of shared space schemes* (**Unknown demographic**). |
| Activities of daily living: There was an awareness from some participants that support from outside the family unit might be complicated, especially where daily routines were already well established. A large range of daily activities could be impacted by VI, and sometimes become additional responsibilities for the caregiver. Many participants discussed the importance of learning skills and knowledge to equip them to best support the person with VI, which had largely just been gained through experience. Some participants felt they would benefit from support in equipping their relative with VI to undertake daily activities. Previous experience of supporting someone with VI could be helpful, and one participant felt that sources of outside support in the home could provide a useful professional distance. | 1. *[I’m] living two lives, or one and a half lives… I have to be involved in a lot of what he does* (**Spouse/partner, moderate VI**). 2. *Friends sort of come and chat and visit and that sort of thing, and they say things like "Is there anything we can do?" but there isn't, you know, really much you can ask anybody to do. We sort of go from day to day and we've got our little routines...* (**Child, severe VI**). 3. *With Macular the carer has to replace the sight lost by the patient. It is knowing what the patient needs and anticipating how to match these needs. The carer has no previous experiences and is not prepared until the need occurs. When Macular is diagnosed the carer needs ‘training’ in their future role* (**Spouse/partner, severe VI**). 4. *Journeys to hospital, journeys for interests, navigating, finding things, clearing up dropped/spilt/broken items. Checking clothing. Reading letters.* (**Spouse/partner, severe VI**). 5. *The carer has to read, process and monitor the material and environment in every area and aspect of the patient’s life. I have to check that nothing is placed dangerously, that medications are in the right place, even that water is in the kettle in case it gets switched on when empty... too many things to list* (**Partner, severe VI**). 6. *I'm more able to cope now, because I'm an expert in my son - and therefore I can cope. But I'm lucky I've had the strength of character to survive… The problem initially was complete lack of knowledge - it's like someone's given you a Martian and said, "Can you look after this Martian?", and you think, well I don't know what Martians do, I don't know how to best help them... As a fully sighted person, I really struggled with what was the best thing to do, and that's in terms of looking after someone who's visually impaired as well as the emotional support that carers actually need, it is information that's the best support I think... you know, all the toys in the toy shop - which ones are gonna work?* (**Child, moderate VI**). 7. *I've never been supported in, for example, becoming more skilled at teaching a blind person how to do tasks that my mother can no longer do because her eyesight became worse… I could benefit from support in how to support a blind person, a visually impaired person - and there doesn't really seem to be anything like that available... teaching me how to teach them* (**Parent, severe VI**). 8. *I knew what to expect a little bit… I was a little bit more prepared – and maybe that was good and maybe that was bad, cos I kind of knew the trouble that might be coming* (**Spouse/partner, moderate VI**). 9. *If someone's too close to that person, if you're trying to get them to learn these new things again, I think it can be quite hard cos you're too close. You know like if you've ever had driving lessons with family or a friend, you just haven't got the patience* (**Spouse/partner, severe VI**). |
| Respite, to give caregivers a break and provide a change of routine. One participant felt they did not require respite care at the moment, but could anticipate a need for “*sanity breaks*” in future. Some participants expressed their concern that if anything happens to them, there would be no alternative provision for support. | 1. *It would be nice if somebody, if you know the RNIB had perhaps volunteers who would say 'Right, we'll come to the house and spend the day with [child], you two go off for the day and you know, go out for lunch, we're here, we'll chat to [child], we'll get her lunch, we'll take her out for a walk', once a week, once a fortnight, once a month, that sort of thing… like a buddy system* (**Child, severe VI**). 2. *It could be just someone to let off steam to… I think that's going to be the main issue, the ability to walk away, to have sanity breaks. I think that's the kind of thing a caregiver needs, it's the respite... it's not overnights and things like that, it's just... an hour…* (**Spouse/partner, moderate VI**). 3. *When I was in hospital, it became apparent the absolute lack of support for my wife, should I not be about. Fortunately friends and neighbours rallied in… but from an official support perspective, there's literally nothing there* (**Spouse/partner, severe VI**). 4. *I need practical support. I dunno, just somebody keeping an eye on her so I can do other things. It's almost like I'm not allowed. Actually there's nothing wrong with her being on her own for a couple of hours, but then I'm led to believe how could I have done that. So I always have to make sure there's somebody else with her… I've got to allow myself a break, I have to say to myself, you deserve this - you're allowed to have a day off* (**Parent, severe VI**). |
| Parents often discussed how schools could better support the caregiver and child together. Insensitive treatment in the school setting could have a long-lasting impact on both the child and caregiver. There was awareness that understanding of VI could vary significantly across the school | 1. *At his primary school, he had a really hard time because the teachers thought I was just being fussy*… *It would have been helpful if they had even opened the books I'd given them from the Nystagmus Network, or even acknowledged that maybe this is a challenge* (**Child, moderate VI**). 2. *The main support needed was dealing with education professionals, trying to educate them in our daughter's needs. In particular to understand that just because she had coping mechanisms did not mean that they did not have to take her needs into account… The primary school were helpful in the sense of taking on board everything we told them, and in the secondary school, the support department were brilliant. But, the actual individual teachers didn't take any notice - well she's coping, so what's the problem? Nobody ever said it in those words... Getting it over to them, yes she looks like she's coping, but that doesn't mean that everything's alright* (**Child, mild VI**). |
| Experiences with QTVIs were somewhat mixed and reflected the unevenness in support and ‘postcode lottery’ mentioned previously. One participant with a positive experience of support from QTVIs praised their ability to provide clear, simple advice to the parent. Another thought QTVI support had been excellent, but differentiated this support for the child from support for the caregiver (while accepting their interdependence). Others had received or were receiving much more perfunctory or limited support | 1. *I learnt a lot from the QTVIs… some really simple things I didn't understand. And what they were great at was explaining things in non-clinical terms for me to understand… I had an early years specialist who would come and work with him at home and who visited him at home, and I had a QTVI that would come in and do the early years development journal for children who are visually impaired. We were very well supported at that time I felt* (**Child, moderate VI**). 2. *The specialist advisory teachers have been absolutely brilliant at giving me pointers on how to help [child] and making sure that [child] is supported… they are brilliant, they really have been fantastic. I'm able to access them and ask them specific questions on anything I feel I don't how best to support [child] with, but their remit is for him, not for me* (**Child, moderate VI**). 3. *Someone came to assess [child] for any additional needs at school… then we got some assistance with yellow film and a tilted desk, and that was it* (**Child, severe VI**). 4. *We know that in order for her to thrive, she's going to need to learn braille, she's going to need all-sector support, and we want to get that in place before she starts school. But you know, you mention things like that to the QTVIs and they're dismissive, because they don't want to provide that extra support or they don't even want to think about an EHC plan just yet, or talk about pre-braille skills*… *Even the Council in terms of the services they give us, they're set up to do the bare minimum to let the children survive but not to give them enough help to thrive, as it were* (**Child, severe VI**). |

# Theme 4: Emotional, relational and psychological challenges of caregiving

| **Description** | **Illustrative quotation(s)** |
| --- | --- |
| Participants emphasised the close interconnection between the wellbeing of the PVI and the caregiver. If the person with PVI is not being adequately supported, this can intensify the strain and distress experienced by the caregiver(s). | 1. *The support we've been getting from government has been pretty poor, and that's not just support for her, it's been support I guess for us... because if they're not giving the support to my daughter, it kind of affects us - we get stressed about things, and we get upset, and it's hard for us…*  *it affects us personally as well* (**Child, severe VI**). 2. *As a caregiver, the thing you focus on is trying to get some relief for the person you're providing care for, because obviously if [child] is more able to cope, more able to sleep, is able to eat, that makes my life easier. And so that's where my focus was. And I'm lucky I didn't go under, but I was very, very depressed at that time...* *If the children are supported, the parents have a slightly easier time of it* (**Child, moderate VI**). 3. *The support for the patient and the support for carers often are one and the same thing… It's very difficult to separate...* (**Parent, moderate VI**). 4. *A caring role is a partnership* (**Parent, severe VI**). |
| Stress was attributed in particular to the complex process of trying to access support for the PVI, and navigating bureaucratic and administrative obstacles. If more support was provided automatically and proactively for the PVI, this would lessen demands on caregivers. Participants implied that often the most valuable kind of support from a mental health point of view would combine practical and emotional support. | 1. *Our needs have really been maintaining our mental health, because it's been so stressful. Dealing with my daughter and her condition's one thing, but then dealing with the paperwork and administration side of getting her the help - you don't really need that* (**Child, severe VI**). 2. *If all these things had happened automatically, I wouldn't have had to do anything at all. Because if the "patient" can do various things for themselves anyway, and will do them providing they know what to do, and when they do it it works, then the carer doesn't have to do a great deal of following up. But if there are difficulties or something hasn't worked, then someone has to make suggestions of what to do next or make phone calls* (**Parent, moderate VI**). 3. *I have to say from an emotional point of view, my health visitor was a better support - they [QTVIs] dealt more with [child] and making sure nursery were aware of how things were, and my health visitor did a lot more of the emotional support of where to get tips from, and disability allowance, and all those kinds of things* (**Child, moderate VI**). |
| Participants discussed the challenge of maintaining a delicate balance between helping and supporting the PVI on the one hand, and ensuring the independence of the PVI on the other hand. | 1. *The child has to take responsibility, I think, at a relatively early age, so they feel empowered by it and not disabled by what's wrong with them* (**Child, severe VI**). 2. *[I would like] some support for [child] to become an independent young man… to have the knowledge that he needs, so that he can know what his difficulties are and be able to have strategies to overcome all of these as best he can* (**Child, moderate VI**). 3. *My wife is a very independent woman, and the last thing she needs is caring… When you do what I do, it's a very fine line being careful of not being overprotective - you get it wrong sometimes but I'm convinced it's the right way of doing it* (**Spouse/partner, severe VI**). 4. *Your basic instinct is to rescue the person you love and make it better for them, but actually that's the worst thing you can do because you take the responsibility away from them. Which means you're left going, 'Well what do I do now? I'm supposed to be rescuing but I can't rescue, I'm not allowed to, what do I do, what's my role?’ That's the thing, what is the parent or carer or partner's role in this scenario?* (**Child, moderate VI**). 5. *It's difficult because you could say, 'Oh, come on, I'll do that', but then you're taking away his independence* (**Spouse/partner, moderate VI**). 6. *They can still go out, they want to be independent, it's just that the eyesight won't allow it. My husband wants to do everything, he doesn't want to be dependent* (**Spouse/partner, severe VI**). 7. *I have to be very very careful that I don't step into what was originally [my spouse’s] province, too much* (**Spouse/partner, severe VI**). 8. *They are going to do things, they are going to fail at things, but that does not give you the right to take over. Cos they will literally hate you for it... Cotton wool is not the answer* (**Spouse/partner, severe VI**). |
| An emotional challenge for the caregiver involved supporting the PVI with their mental health and wellbeing. | 1. *[My daughter’s] condition has affected her mental health and we have supported her with this… I answered this survey as I feel that we will always need to be extra supportive of her due to her visual impairment, despite her amazing resilience!* (**Child, moderate VI**). 2. *One thing is how to help [my mother] to cope with the emotional distress of having lost more sight and having worse vision, because she's extremely stressed by having lost more sight, you know, quite suddenly* (**Parent, severe VI**). 3. *The hardest thing is knowing the best way to support / prepare my son to cope with losing his sight* (**Child, mild VI**). 4. *We tried to get [my child] cycling, we really tried very hard, but it's just dangerous. So there's a lot of things, as kids grow up and wanna do them, he is unable to do... and that's hard* (**Child, moderate VI**). |
| While the PVI and caregiver may have closely intertwined experiences, some caregivers also focused on the need to consider the wellbeing of the caregiver as firmly distinct from that of the PVI, and the need for support to help caregivers cope with the shock, sadness, guilt, loneliness and fatigue they may feel. Particularly when visual impairment developed more suddenly, it could cause considerable shock for the caregivers and lead to abrupt changes in their lifestyle. | 1. *A minority of people don't understand that the family need to express their emotions. And they don't want to do it to their partners or their loved ones, because we feel they're carrying enough of their own emotions… It's still difficult now, lots of times... but I can maybe deal with it a bit better, or give myself the tools… It's made me a better person, I can do anything, because I've had to learn to adapt like that. But there's so many highs and lows... I've heard lots of people explaining that they go through similar things, and they feel guilt…* (**Spouse/partner, severe VI**). 2. *I said to my partner [with VI], ‘I'm not living it the way you're living it... It must be horrific for you, but I'm also living it a different way and trying to hold everything together... We live it in a totally different way’* (**Spouse/partner, severe VI**). 3. *I think it's more... not so much practical help, but the one thing that I think people miss is the emotional impact, and the tiredness that goes on. It is very very tiring. I think [what caregivers need] are a few basic phrases and key points. You know, it is ok to be scared. They [the PVI] will get upset with you, but it's not you they're upset with. You are gonna cry, you are gonna laugh... it's those key things, that basically goes: it's going to seem like you're alone at the moment, but you're not. It's the emotional support that basically says, look, there are people around... Things in your life will have to change. But that doesn't mean you have to give up… The diagnosis is going to shatter their lives, the caregiver's as well as the actual patient's. The caregiver needs to know, you are going to get it wrong* (**Spouse/partner, severe VI**). 4. *It's been a huge shock to the system really*… *She's totally reliant on us to do everything for her, which we're very happy to do - we're her parents - but it's impacted our life enormously, as you can imagine. For instance we haven't been on holiday for 10 years now, because we just can't leave her* (**Child, severe VI**). |
| Parents discussed how their parental anxieties could be magnified by issues linked to VI. Some parents recognised retrospectively that they might have benefitted from more structured psychological support such as counselling, although felt that it was not the right time to explore difficult feelings when they were so busy looking after their child. | 1. *You're always concerned, you've got that concern at the back of your mind at all times. But then I imagine that if you spoke to any parent about any child, with or without a disability, they would have that feeling at all times. I suppose it's just focussed a bit more when you've got a person in the household with a problem...* (**Child, severe VI**). 2. *I think I did ask to have some counselling, but it was such a massive wait and then it would only have been six sessions, and then you just think actually, really what's it going to do? I just kind of gave up. There are only so many fights you can do. And I was fighting for [child’s name] but I wasn't fighting for me, because I just didn't have the energy* (**Child, moderate VI**). 3. *I was offered some counselling at the same time... I don't know why I didn't do it. I probably didn't want to face it, to be honest, with everything that was going on at the time* (**Child, moderate VI**). 4. *What I'll say is we are focusing on trying to get all the support that we think [our child] needs. So that's our focus. We haven't really stopped to focus on us... It hasn't really hit home yet. We're just keeping ourselves busy at the minute. I'm half expecting it to catch up on us... but yeah I don't want to think about that. I just want to keep positive and keep looking forward* (**Child, severe VI**). |
| Several participants suggested they would benefit from more emotional support. What participants termed ‘emotional support’ often chimed closely with the idea of a regular, low-level contact, rather than a more formal psychological intervention like counselling. | 1. *Information on basic support emotionally for family members who have sight loss or are experiencing sight loss* (**Unknown demographic**). 2. *Certainly [there's been] no emotional support, support to check that I was coping as a human - no, I certainly didn't get any of that… Even if they just phoned. It doesn't have to be a home visit, just somebody to check in, just once a month, with the carer, to check I was surviving* (**Child, moderate VI**). |
| The experience of caregiving was often misunderstood by others, and some caregivers worried about burdening their family members and friends. Therefore, often meeting peers in similar situations could create a sense of belonging. Faith-based organisations could also offer a sense of emotional support. | 1. *Because as a couple we're quite independent, and we always seem to look like we're managing, everyone just assumes that we are managing* (**Spouse/partner, severe VI**). 2. *We just think it's, sort of, a bit of a nuisance for people. I'm sure people would say 'Oh don't be silly, it's not a problem' but you just feel it might be* (**Child, severe VI**). 3. *It can be reassuring to learn from others… and hear stories that show that people cope* (**Child, mild VI**). 4. *I went to a religious place, when I was feeling overwhelmed… the person said to me, 'Do what you're doing that's good, and you're going to be repaid from a higher force’* (**Parent, severe VI**). |

# Theme 5: Interpersonal and social support

| **Description** | **Illustrative quotation(s)** |
| --- | --- |
| Meeting other caregivers or supporters of PVI had been, or was imagined to be, a transformative experience for many caregivers, allowing them to both provide support to peers and benefit from support themselves. Long-term immersion in a group setting attended by PVI and caregivers was sometimes considered a helpful way to help resolve questions and concerns as they arose. Some participants even suggested they would find it a useful ‘lay’ alternative to group therapy or counselling. | 1. *I feel there should be a lot more support for the partner/family. More places to share and talk about our experience. We try to stay strong for our family members to help their well-being. This then impacts the person offering the support. We feel a lot of guilt for needing to talk as though we are being disloyal - I have heard this mentioned on so many occasions* (**Spouse/partner, severe VI**). 2. *[I would like] someone to talk to who was in the same position as myself, but was also positive and not negative!* (**Child, severe VI**). 3. *[It would be helpful] meeting other carers of similar age and experiences* (**Child, moderate VI**). 4. *We meet every two weeks anyway, so I suppose without realising if I needed anything or had any questions, [the support] is already there* (**Spouse/partner, severe VI**). 5. *It’s nice to be able to talk to other people in the same kind of situations, even getting little tips and stuff like that. Or even if somebody goes, 'Oh I'm struggling with this'. And you can relate to it, or you can give advice back to them, saying 'This is what I found helpful'* (**Spouse/partner, severe VI**). 6. *It’s important to have somewhere where people can sit and talk... I've been going to a community place, and once one person starts talking, suddenly everyone starts talking* (**Parent, severe VI**). 7. *It would be lovely to know there was some sort person I could contact or group who I could join to just chat about our personal experience and if there were ways I could better support my husband* (**Spouse/partner, severe VI**). 8. *I think if her sight goes to the extent that she really is, or we're both, struggling, and having been aware of how much struggle it can be to be a carer, then to have support from a group where you can talk to people who are going through the same things you are, the same worries you've got, that is really important* (**Spouse/partner, severe VI**). 9. *Probably somebody who had some knowledge of the effect of having someone with different needs (not necessarily eyesight ones) who you could talk to and say, 'This is what's happened, this is how we feel' and talk it through with… Counselling, I dare say, but maybe not under that title* (**Child, mild VI**). 10. *I think if people can let off steam to friends or people in the same boat - peers - I think that's as good, or better, than a counsellor* (**Spouse/partner, moderate VI**). |
| Finding the appropriate group in terms of shared experience was not always a guarantee of support, and some participants pointed to how different support groups inevitably had their own unique personalities and atmosphere. The nature of VI and the many different conditions associated with VI could make finding people in comparable situations difficult. | 1. *The first group was a waste of time… but [another branch] was* *friendly, cooperative and welcoming, which enables people to help each other. Group interaction gives you a feeling of wellbeing, that's what it's all about* (**Spouse/partner, Moderate VI**) 2. *I think it's difficult to get support with peers, because it's such a low incidence condition, so to find a group of people who have a similar condition to you is difficult... That's how we ended up with Nystagmus Network, we went to their annual meeting a couple of times... It was great for [my child] to mix in with other kids who had nystagmus. For night blindness, I suppose the gap is... you can't make a group for people where there isn't a group of people, if you know what I mean. So it's quite lonely sometimes. Group supports are difficult to find… as there is such a range of visual impairments* (**Child, Moderate VI**) |
| There was awareness among participants that social support groups are not always easy to find. Some had come to groups through serendipity, and suggested that buddy systems whereby newer caregivers are paired with more experienced caregivers could be helpful, recognising and responding to the gap in connecting to the relevant services | 1. *People need help, and they're not going to get help unless they are told about these groups and come to them and meet other people like themselves. The more we get, the more we can help. But unless they're sent to us, we can't do anything… If they come to us, they'd be a lot happier. They'd see other people like themselves, as well. They think they're on their own, you see…* (**Sibling, Moderate VI**) 2. *A carer that's experienced will be linked up with someone in the district who's coming into the caring function... because there is this realisation that there is this gap of how to get help for the carer* (**Spouse/partner, Severe VI**) |
| Participants discussed the importance of online communities and support through social media; for example in the form of a ‘caregivers only’ forum on charity websites, a Facebook group for family members, or an e-newsletter for caregivers. | 1. *We have started a UK based Facebook group to support parents of VI children* (**Child, Severe VI**) 2. *I think a platform for people supporting people with sight loss, perhaps under the Macular [Society] website, a section which is for caregivers, something like that might be worthwhile. I think that's what missing from my point of view, the place to go for people to give you some ideas and information* (**Spouse/partner, Moderate VI**) 3. *[I’m in] a Facebook group, a safe and secure place for families to talk in confidence… The thing it helps the most with is isolation. Being able to speak to other people, not in exactly the same situations but similar situations. Being able to go to someone who's living it 24/7... We live it all day... And little tips, that parents give to other parents, about education, PIP forms, being able to share all those tips and tricks: ‘I'm new to this, with a new baby... how do other parents encourage them to play, to be more independent when they get to that stage?’ Little tips but they make a massive impact on that person; they don't have to wait a year, two years to get the solution - there's somebody who lived it two or three years ago, or months ago, and they are sharing their experience and they're giving their knowledge down… You just think, I'm not alone* (**Spouse/partner, Severe VI**) 4. *Any support I get for supporting my husband I get from family and friends or from social media groups. Very often I have been made to feel that I have no right to struggle as my husband’s life is so much harder* (**Spouse/partner, Severe VI**) 5. *Receiving information on a regular basis, maybe a sort of e-newsletter or something, directed at people who perform this role, that could be quite interesting... which would also be saying you know, have you thought of doing this, or this is being done somewhere, or some people have found this to be helpful, maybe you'd think about doing this, and that sort of thing...* (**Parent, Moderate VI**) |
| Social media groups can overcome the financial and mobility barriers to attending group meet-ups, although in an ideal world it was thought that accessible, funded in-person meetings for caregivers could be highly beneficial, especially at the local level. | 1. *Lots of events are just for people with sight loss. I'd love to set something up for family members, where you all go into a big room, you all split off. Everybody comes together, you have a day together, then you go in your own little groups. For example, siblings talk to siblings, the partners can be somewhere else... So that people can just say everything out loud* (**Spouse/partner, Severe VI**) 2. *[It would be good to] have something in place like a safe space for people to talk, maybe in local authorities, maybe that you could join, once a year you had a reunion* (**Spouse/partner, Severe VI**) 3. *I think if there was more local group... There might be others and people within your area that you at least vaguely know, or people understand your local area even if you don't know the people* (**Spouse/partner, Severe VI**) |
| Another important aspect of support from others, especially for parents, emerged when they felt people were understanding about the condition of the person with VI. For example, several parents had had experiences of misunderstanding from teachers about their child’s condition. Some participants believed that this lack of understanding and empathy from others could be explained by the fact that VI is not always obvious to others, even people close to the caregiver and PVI. | 1. *[The thing that would help most would be] if people were more educated about [my partner’s] condition* (**Spouse/partner, Severe VI**) 2. *[I would like] people to understand the impact of a disability that is not immediately apparent by looking at the person. This would take the stress off* (**Child, Severe VI**) 3. *A teacher in class told him to stop doing that with his eyes because 'he looked weird'… you can imagine what that does to a child* (**Child, Moderate VI**) 4. *A lot of the visual issues aren't immediately apparent* (**Child, Mild VI**) 5. *[Vision impairment is] hidden and hideable* (**Spouse/partner, Severe VI**) 6. *We'll go round family, and they'll just have the lights on low, they won't put the main light on... It's things like that, people just assume that if you have a visual disability or if you've got a cane, one you're totally blind and you can't see anything at all, and two is the lighting; it's not normally known that, 'Oh actually, these people need bright lighting'* (**Spouse/partner, Severe VI**). |

# Theme 6: Caregiver identity and recognition

| **Description** | **Illustrative quotation(s)** |
| --- | --- |
| Many participants felt uncomfortable with the term ‘caregiver’, finding it was not relevant to them, too extreme, or even that it was disempowering or derogatory, positioning the person with VI as dependent. | 1. *There is a boundary, I think, between support and care, and at the moment all I'm providing is support, really… Certain situations I suppose, yeah, like if we're out and it's dark and we're somewhere unfamiliar, she'll sometimes grab my hand or say to me, 'I can't see' and I slow down a bit, but that's support, not caring. I don't have to do anything for her activities of daily living at all, she's totally independent... I don't feel I'm a carer, perhaps a supporter* (**Spouse/partner, Severe VI**) 2. *When you say caregiver, you kind of imagine giving care to, say, someone who, it sounds silly but, doesn't normally need it; maybe you're caring for an adult or you're caring for a elderly parent or caring for a neighbour? When you say caregiver, because you kind of look after your children anyway... it did seem really alien and really strange, and I don't think I would have felt that if I was talking about an older person. Or even if my child was a lot older, I don't think I would have felt so weird… It doesn't seem right, but then if you think about the definition of it then I guess we are caregivers. But ‘caregiver’ is quite intimidating; something else would be much wordier, but much softer* (**Child, Severe VI**) 3. *The word 'carer' does not properly describe what I do because she is a very independent person who is determined to make the most of the little vision she has. I don't like the word 'caregiver' that you have invented, what's wrong with 'carer' if it is appropriate?* (**Spouse/partner, Severe VI**) 4. *Caregiver is the worst way of putting it… I am a facilitator… ‘Care' is just the wrong word, because it says you're doing something for somebody. No, my role is to assist somebody to do something themselves* (**Spouse/partner, Severe VI**)*.* 5. *I don't feel I am a carer, as my mother manages very* *well* (**Parent, Moderate VI**). |
| Many participants saw looking after a child or supporting a spouse or parent as natural. | 1. *I don't particularly class myself as a carer in respect that I'm a mum and that responsibility to me means I care for my VI child in the same way I care for my non VI child in that I do as much for each of them as they need me to* (**Child, Moderate VI**) 2. *I just feel that I'm a mum really, and that's what I do… I associate caregiver with someone looking after someone older… that's my personal view. I think I'm just doing what a mum would do* (**Child, Moderate VI**) 3. *Although I'm considered a 'caregiver' essentially I'm a husband so what my wife needs I try my best to provide* (**Spouse/partner, Severe VI**) 4. *People seem to think that I'm a hero doing what I'm doing. No, I'm a husband who is honouring an agreement that I made. We do it because we care, all people who care - but it's just that, I guess, sometimes, I guess my life is defined by her condition as well, which means there are certain things that I know I can no longer do and have access to* (**Spouse/partner, Severe VI**) 5. *I'm a wife, and a mother… I don't want to be called a carer… I don't want to be labelled as a carer. In some instances people do, but I don't like the name* (**Spouse/partner, Severe VI**) 6. *We vowed that we would look each other, and that bond is reciprocated... So I didn't see myself as [my wife’s] carer. I've begun to realise I'm a carer as more and more duties have been passed on. But she looks after me as well, and cares for me...* (**Spouse/partner, Severe VI**) |
| Some participants reluctantly accepted the term ‘caregiver’ or ‘carer’, or acknowledged the difficulty of finding a suitable alternative. | 1. *Caregiver, no… but what else would you call it?* (**Child, Severe VI**) 2. *I suppose that's what I am, but I don't like to see myself like that. Predominantly, first, we're husband and wife… it's what you do when you get married, for better for worse so to speak. So I don't really like to think of myself as a caregiver, though I am in a way* (**Spouse/partner, Severe VI**) 3. *I don't really regard myself as a carer for my mother, but I do help her with things... I suppose in a very loose sense of the word, I could call myself a carer. I can't think of an alternative term apart from family member or friend* (**Parent, Moderate VI**). 4. *I'm not that comfortable with it [the term caregiver], because it depends what it means, but I guess there's a lot of people who do more than I do who are not tremendously comfortable with it, they just do it... But again, you have to give some kind of label if you're trying to talk about these people collectively, you have to somehow describe who they are* (**Parent, Moderate VI**) |
| Some participants found the carer/caregiver label unproblematic, and even a useful term when liaising with support services. | 1. *I'm definitely a carer, or caregiver - that's unambiguous. Personally, sometimes I have to say I'm a carer, in the context of social services or whatever... you know, you just accept it. It's not always cut and dry, it's not always straightforward* (**Parent, Severe VI**). 2. *I think caregiver is better than actual carer, cos if you're a carer you're with them most of the day… While I wasn't with her most of the day, but I was there when she needed the help* (**Sibling, Moderate VI**). 3. *The term doesn’t bother me either way, I suppose I am a caregiver* (**Spouse/partner, Severe VI**). |
| Some participants also discussed how recognition by the self and from others of the efforts involved in being a caregiver,  beyond what might be reasonably expected from a relative, could be an important step in seeking out and connecting to support services. This would involve a shift in mindset away from seeing caregiving as a given, or automatically expected. | 1. *One of the other things is we, as a community, when we do these roles, we don't really see ourselves as carers. There's an automatic understanding that we'll do this... when I look after my mum for example, people wouldn't see me as a carer, would they? They'd see me as a daughter. No matter how much I'm doing beyond the call of duty, they wouldn't see me as a carer. So, unfortunately, lots of people won't then claim for things they're entitled to, because if you don't own that hat of a 'carer', then when you see the word you don't relate it to you, necessarily, do you, you just say, 'I'm not a carer am I', but you are! But they don't know that… I suppose I'm talking about that community of people. I don't necessarily see myself as a carer either. I just think it's something I just... do!* (**Parent Severe VI**) 2. *There's a lot of mixed messages out there, like you've got to be doing a certain amount of carer’s role before you can get help yourself... And if there are bits and pieces available for carers, then you feel a bit guilty for taking up that support, as you're not doing this role because you want something - and that's the change of mentality isn't it, to not feel guilty* (**Parent, Severe VI**) 3. *It can be more than a human being can kind of adequately cope with. It's a lot to put on anybody… there is no recognition that actually people are even doing these tasks, let alone need some support* (**Child, Moderate VI**) 4. *[It would help] not being expected to do the caring* (**Partner/spouse, Severe VI**) |
| A significant issue for many caregivers is a lack of official recognition of their role. This can cause practical problems when dealing with healthcare professionals, and has implications for the support caregivers themselves receive. | 1. *You're not a nominated person, the caregiver does not exist in the system - that's the main problem, isn't it... If I was a guide dog I would exist, because of the Guide Dogs Association, but because I'm not a guide dog I don't exist. I have no status whatsoever… I understand the rules and regulations… but at the present moment you can't be recognised as a person who needs to know... You could have a card, and when one person has one half, the other has the other half, and if you could match them, then 'Yes we can tell you'. Some sort of link, to get round the bureaucracy of privacy… because there is no such thing as a caregiver in the real world* (**Spouse/partner, Moderate VI**) 2. *I don't think I am officially recognised as a care giver by the health services, despite attending key appointments (eg when the certificate of VI was registered). There has been no mechanism for this to be noted, therefore no recognition that I or anyone else might need any assistance… It might be very helpful for services to know that there is a nominated person and who that might be. Obviously they would have to give their consent, but for that to be acknowledged would be quite helpful* (**Parent, Moderate VI**). 3. *[We need] recognition that caring by a family member (although given with love) has significant financial impact for the carer both in the ability to earn now and to prepare for old age with savings and pensions* (**Parent, Severe VI**). |
